# Assessing risk of bias in human environmental epidemiology studies using three tools: different conclusions from different tools

**DOI:** 10.1186/s13643-020-01490-8

**Published:** 2020-10-29

**Authors:** Stephanie M. Eick, Dana E. Goin, Nicholas Chartres, Juleen Lam, Tracey J. Woodruff

**Affiliations:** 1 Program on Reproductive Health and the Environment, Department of Obstetrics, Gynecology and Reproductive Sciences, University of California, San Francisco, USA; 2 Department of Health Sciences, California State University, East Bay, Hayward, CA USA

**Keywords:** Risk of bias, Systematic review, Quality assessment, Critical appraisal, Evidence evaluation, Risk assessment

## Abstract

**Background:**

Systematic reviews are increasingly prevalent in environmental health due to their ability to synthesize evidence while reducing bias. Different systematic review methods have been developed by the US National Toxicology Program’s Office of Health Assessment and Translation (OHAT), the US Environmental Protection Agency’s (EPA) Integrated Risk Information System (IRIS), and by the US EPA under the Toxic Substances Control Act (TSCA), including the approach to assess risk of bias (ROB), one of the most vital steps which is used to evaluate internal validity of the studies. Our objective was to compare the performance of three tools (OHAT, IRIS, TSCA) in assessing ROB.

**Methods:**

We selected a systematic review on polybrominated diphenyl ethers and intelligence quotient and/or attention deficit hyperactivity disorder because it had been endorsed by the National Academy of Sciences. Two reviewers followed verbatim instructions from the tools and independently applied each tool to assess ROB in 15 studies previously identified. We documented the time to apply each tool and the impact the ROB ratings for each tool had on the final rating of the quality of the overall body of evidence.

**Results:**

The time to complete the ROB assessments varied widely (mean = 20, 32, and 40 min per study for the OHAT, IRIS, and TSCA tools, respectively). All studies were rated overall “low” or “uninformative” using IRIS, due to “deficient” or “critically deficient” ratings in one or two domains. Similarly, all studies were rated “unacceptable” using the TSCA tool because of one “unacceptable” rating in a metric related to statistical power. Approximately half of the studies had “low” or “probably low ROB” ratings across all domains with the OHAT and Navigation Guide tools.

**Conclusions:**

Tools that use overall ROB or study quality ratings, such as IRIS and TSCA, may reduce the available evidence to assess the harms of environmental exposures by erroneously excluding studies, which leads to inaccurate conclusions about the quality of the body of evidence. We recommend using ROB tools that circumvents these issues, such as OHAT and Navigation Guide.

**Systematic review registration:**

This review has not been registered as it is not a systematic review.

## Background

Systematic review methods are becoming increasingly recommended and used to inform environmental health decisions [1–4]. Application of these methods supports greater transparency in the scientific basis and judgments behind public health decisions [5]. Well-conducted systematic reviews therefore result in improved consistency of evidence with less bias [5]. Systematic reviews have been adapted from clinical medicine, where data is largely derived from randomized, controlled trials [6]. However, environmental health systematic reviews used to inform the relationship between exposures and adverse health outcomes must typically rely on data from human observational studies due to ethical considerations [5]. The University of California, San Francisco (UCSF), developed the Navigation Guide, which was the first systematic review methodology aimed to evaluate the evidence linking environmental exposures to adverse health outcomes [5]. Since then, different systematic review methods have been developed and implemented by various US authoritative bodies and agencies including the National Toxicology Program (NTP)’s Office of Health Assessment and Translation (OHAT) [7], Environmental Protection Agency (EPA)’s Integrated Risk Information System (IRIS) [4], and by EPA under the Toxic Substances Control Act (TSCA) [8] to inform environmental health decisions that have major implications for public health. An important benefit of these tools is that they are specifically designed for evaluating studies of environmental exposures, thus eliminating the translation required from more generic tools applied in clinical medicine [4].

The evaluation of the risk of bias (ROB) of the included studies is a key component in a systematic review to assess the validity of the studies which are the basis of the conclusions of the review. ROB is a measure of internal study validity that reflects features of a study’s methodological design, conduct, or analysis that can lead to a systematic under- or over-estimation of the true effect the study aims to estimate [9].

In clinical systematic reviews, the Cochrane Collaboration’s ROB evaluation tool is widely accepted and used [6]. However, as environmental health assessment evidence often consists of observational human studies of exposure to evaluate harms, this tool cannot be applied directly. While there are multiple tools currently in use to assess the ROB in human observational studies, there is a lack of consensus over advantages of any one single instrument over another for use in systematic review or guideline development [10]. There is also limited empirical evidence about how well individual questions on existing and new ROB instruments assess the exposure-outcome relationship [11]. Selection of the items for a ROB tool should, at a minimum, be informed from domains with empirical evidence in randomized trials and conceptual considerations [12, 13]. The lack of agreement on the most appropriate method to use to evaluate the ROB of observational design studies can make both systematic reviews and the assessments and guidelines they inform challenging to evaluate and interpret [14]. Furthermore, the application of different tools to assess the ROB of a single observational study has been shown to lead to different conclusions [15, 16].

Although there is no consensus on which is the best tool to assess ROB in observational studies, several tools have been developed from the tools used to evaluate clinical interventions [5, 7, 9]. Methods such as the Navigation Guide rely on the evaluation of individual domains, which provides a structured framework within which to make qualitative decisions on the overall quality of studies and to identify potential sources of bias [17]. In contrast, several methods include an “overall quality score” to assess ROB of individual studies. Recent studies have suggested that it is inappropriate to use these “overall quality scores” [14], as they have been shown to inadequately distinguish between studies with a high and low ROB in meta-analyses [18, 19]. Importantly, there is a lack of empirical evidence to demonstrate how individual items should be weighted when creating “overall quality scores” [17, 18, 20]. Thus, overall ROB scores can lead to biased evaluations of the literature, as several systematic review methods propose that studies that receive poor ratings for overall study quality are excluded from the overall body of evidence [20]. Overall quantitative scores have also been criticized and recommended against by the National Academy of Sciences (NAS), as they can be a poor reflection of overall study quality [21, 22]. While there is inevitably variation in the internal validity and ROB across individual studies, the most appropriate method to exclude studies is based on predefined exclusion criteria, as opposed to an arbitrary rating of the evidence, which is often based on a limited number of domains with minimal empirical basis [7].

There is a need, therefore, to understand how different tools designed to assess the ROB of human observational studies compare to one another in terms of their reliability and usability, in particular, those that are used by government agencies due to their importance in policy and regulatory decision-making. Further, it is important to understand if differences between these ROB methods influence the overall conclusions of a systematic review, which could affect conclusions about the evidence and subsequent public health recommendations and action by decision-makers. Therefore, the objective of this study was to compare the performance of the different ROB tools used by NTP’s OHAT [7], US EPA’s IRIS program [4], and by US EPA under TSCA [8] in a case study relevant to human environmental epidemiology. Specifically, we aimed to assess the usability of each tool by calculating the mean time to conduct the ROB assessment, evaluate inter-rater reliability for each tool, and determine the impact of the ROB ratings for each tool on the conclusions with respect to the overall quality of the body of evidence.

## Methods

### Case study

We compiled a diverse team of researchers with expertise in reproductive epidemiology, environmental health, systematic review, and public health, all with doctoral degrees. Two reviewers (SME, DEG), applied the OHAT, IRIS, and TSCA systematic review tools to 15 human epidemiologic studies on polybrominated diphenyl ethers (PBDEs) and intelligence quotient (IQ) and/or attention deficit hyperactivity disorder (ADHD) from a previously completed systematic review [23]. We selected this review because it applied the Navigation Guide framework to assess ROB, a framework that has been empirically demonstrated on six case studies to date [23–28] as well as endorsed by the NAS [29]. In particular, the PBDE and IQ/ADHD case study was critically evaluated by the NAS, concluding that there was no evidence of bias in the assessment [3]. However, we acknowledge that there is likely no tool that can provide a fully objective measure of ROB. Although numerous tools exist to assess ROB, we focused on the OHAT [7], IRIS [4], and TSCA [8] tools in our review because they are currently in use by the US government (NTP, EPA), and routinely applied for assessing risks from environmental chemicals. These tools all evaluate different constructs related to validity, including elements of ROB as well as study quality (Table 1). Therefore, for consistency, we subsequently use the term ROB when describing these tools and how they were applied.

**Table 1:** Description of tools used to assess risk of bias

Program or agency	Tool	Assessment process	Domains assessed	Number of questions	Available answer options
NTP	OHAT	Risk of bias, internal validity	Selection bias	1	Definitely low ROB; probably low ROB; probably high ROB; definitely high ROB
			Confounding bias	1	Definitely low ROB; probably low ROB; probably high ROB; definitely high ROB
			Attrition/exclusion bias	1	Definitely low ROB; probably low ROB; probably high ROB; definitely high ROB
			Detection bias	2	Definitely low ROB; probably low ROB; probably high ROB; definitely high ROB
			Selective bias reporting bias	1	Definitely low ROB; probably low ROB; probably high ROB; definitely high ROB
			Other bias	1	Definitely low ROB; probably low ROB; probably high ROB; definitely high ROB
EPA	IRIS	Study evaluation	Exposure measurement	1	Good; adequate; deficient; critically deficient
			Outcome ascertainment	1	Good; adequate; deficient; critically deficient
			Participant selection	1	Good; adequate; deficient; critically deficient
			Confounding	1	Good; adequate; deficient; critically deficient
			Analysis	1	Good; adequate; deficient; critically deficient
			Selective reporting	1	Good; adequate; deficient
			Sensitivity	1	Adequate; deficient
			Overall study confidence	1	High; medium; low; uninformative
EPA	TSCA	Data quality criteria for epidemiological studies	Study population	3	High; medium; low; unacceptable
			Exposure characterization	3	High; medium; low; unacceptable
			Outcome assessment	2	High; medium; low; unacceptable* *Unacceptable not available for one question
			Potential confounding/variable control	3	High; medium; low; unacceptable* *High and unacceptable not available for one question
			Analysis	4	High; medium; low; unacceptable* *High and low not available for two questions *High and unacceptable not available for two questions
			Biomarker selection and measurement	7	High; medium; low; unacceptable *High and unacceptable not available for one question *Unacceptable not available for three questions
			Overall study confidence	1	Sum of weighted scores/sum of metric weighting factor
UCSF	Navigation Guide	Risk of bias, internal validity	Source population representation	1	Low; probably low; probably high; high
			Blinding	1	Low; probably low; probably high; high
			Exposure assessment	1	Low; probably low; probably high; high
			Outcome assessment	1	Low; probably low; probably high; high
			Incomplete outcome data	1	Low; probably low; probably high; high
			Selective outcome reporting	1	Low; probably low; probably high; high
			Confounding	1	Low; probably low; probably high; high
			Conflicts of interest	1	Low; probably low; probably high; high
			Other	1	Low; probably low; probably high; high

Abbreviations: *NTP* National Toxicology Program, *OHAT*, Office of Health Assessment and Translation, *EPA*, Environmental Protection Agency, *IRIS* Integrated Risk Information System, *TSCA* Toxic Substances Control Act; *ROB* risk of bias, *UCSF*, University of California, San Francisco

### Risk of bias tools

#### OHAT

The OHAT handbook describes a tool for evaluating the ROB for both human and animal studies [7]. The OHAT tool consists of an overall set of questions, of which certain questions are relevant for human versus animal studies. The OHAT tool is domain-based and asks seven questions relevant for human studies which cover six possible sources of bias: participant selection, confounding, attrition/exclusion, detection, selective reporting, and other sources [7]. The detection bias domain includes two questions, one on exposure characterization and the other on outcome characterization. Answer options for each question are the ratings “definitely low,” “probably low,” “probably high,” and “definitely high” ROB. The OHAT tool does not apply an overall rating for each study. Additionally, OHAT instructs that studies should not be removed from consideration of the overall body of evidence as a result of “probably high” or “definitely high” ratings.

#### IRIS

The IRIS tool [4] is an adaption of the risk of bias in non-randomized studies of interventions (ROBINS-I), which evaluates the ROB predominantly in non-randomized studies investigating the comparative effectiveness (harm or benefit) of clinical interventions [30]. The IRIS method states that it has modified this tool for applications to assess population health risks resulting from exposures to environmental chemicals. IRIS states that the tool is designed to evaluate study quality or study utility, which encompasses multiple issues including ROB, study execution, study sensitivity, and reporting of results. The IRIS tool is a domain-based tool and includes seven questions spanning possible sources of bias: participant selection, confounding, selective reporting, exposure measurement, outcome ascertainment analysis, and sensitivity. For each question, answer options are the ratings “good” (indicative of a low ROB), “adequate” (indicative of a probably low ROB), “deficient” (indicative of a probably high ROB), “critically deficient” (indicative of a high ROB), and “not reported.”

In addition to the seven sources of bias, the IRIS tool also includes a question on “overall study confidence,” which relies on interpreting the ratings from all domains and on reviewer interpretation, as opposed to utilizing a weighted average or other numeric equation. The IRIS guidelines say that overall study confidence “will be based on the reviewer judgments across the evaluation domains for each health outcome under consideration, and will include the likely impact the noted deficiencies in bias and sensitivity, or inadequate reporting, have on the results.” This can be interpreted as “high” if all domains are rated “good,” “medium” if all domains are rated “adequate” or “good,” “low” if one or more domains are rated “deficient,” and “uninformative” if any domain is rated “critically deficient.” However, the IRIS tool permits subjective reviewer judgment when assessing overall study confidence, and alternate approaches may be possible. Therefore, while we used the instructions explicitly to rate the ROB, in an effort to examine the robustness of our original findings, we also conducted a sensitivity analysis to determine if the overall study confidence rating would vary with alternative guidance. Specifically, we rated the overall study confidence to be “high” if none or one of the domains was rated as “deficient,” “medium” if two of the domains were “deficient,” and “low” if three or more domains were “deficient” or “critically deficient.” As with the original rating process, studies deemed “low” or “uninformative” overall study quality are removed from the overall body of evidence.

#### TSCA

The TSCA ROB tool [8] is presented as a ROB evaluation tool that includes quantitative scoring. The TSCA tool is domain-based and employs a quality-based scoring system which includes 22 metrics (questions). These metrics are grouped into 6 possible sources of bias: study population, exposure characterization, outcome assessment, potential confounding/variable control, analysis, and other. These metrics conflate ROB concepts with study quality domains (e.g., how well the study is reported). Answer options include “high” (a quantitative score of 1; indicative of a low ROB), “medium” (score of 2; indicative of a probably low ROB), “low” (score of 3; indicative of a probably high ROB), and “unacceptable” (score of 4; indicative of a probably high ROB). Not all answer options are available for each question. For example, the question assessing co-exposure confounding requires reviewers to choose “medium” or “low” only and it does not allow choosing “high” and “unacceptable” (Table 1).

The TSCA method identifies key or “critical” metrics and gives them a higher weight than other metrics within the same domain. Examples of critical metrics include participant selection, temporality, and covariate adjustment. Critical metrics are assigned a weighting factor that is twice the value of the other metrics within the same domain and the non-critical metrics are assigned a weighting factor of half the weighting factor assigned to the critical metrics within each domain. The TSCA method states that “critical metrics are identified based on professional judgment in conjunction with consideration of the factors that are most frequently included in other study quality/risk of bias tools for epidemiologic literature” [8]. However, there is no documented reference for this decision process and the TSCA method has not gone through peer review. If the response to any individual question is “unacceptable,” the overall study quality is automatically rated as “unacceptable.” Studies deemed “unacceptable” are removed from the overall body of evidence. Each study is subsequently given an overall score, calculated by multiplying the score for each metric by a weighting factor and then dividing by the sum of the weighting factors (see Supplemental Material).

Detailed instructions for making ROB determinations provided by each tool are provided in the Supplemental Material (see “Instructions for making risk of bias determinations using OHAT framework,” “Instructions for making risk of bias determinations using IRIS framework,” and “Instructions for making risk of bias determinations using TSCA framework”). Instructions applied in this case study were taken verbatim from the methods documentation of each tool. The TSCA tool applied in the risk evaluation for 1,4-Dioxane was applied here.

#### Comparison of domains assessed across tools

The Navigation Guide, OHAT, IRIS, and TSCA tools all assess bias due to exposure and outcome measurement, study population, and confounding. The Navigation Guide is the only tool to include conflicts of interest as a separate ROB domain. In contrast, TSCA is the only tool to consider biomarker selection and measurement. IRIS and TSCA are the only tools that include indicators of overall study quality.

### Application of risk of bias tool and statistical analysis

Prior to applying the different tools, the two reviewers (SME, DEG) completed training (approximately 4 h) on assessing ROB in epidemiology studies with a systematic review expert (JL). Trainings included a broad overview on assessing ROB and specific clarification on the application of each tool.

Each tool asks at least one question regarding confounders or covariates retained in adjusted models (see “Instructions for making risk of bias determinations” in the Supplemental Material). Therefore, we made pre-specified list of important confounders (Tier 1) and other potentially relevant confounders (Tier 2) from the previously completed systematic review on PBDEs and IQ and/or ADHD [23]. Confounders were identified by individuals with subject matter expertise on PBDEs and IQ and/or ADHD. This list included Tier 1 (HOME inventory, maternal age, maternal education, marital status, maternal use of alcohol during pregnancy, maternal depression, household income/poverty, gestational exposure to environmental tobacco smoke, child sex, exposure to other neurotoxic agents) and Tier 2 (birth weight or gestational age, number of children in the home, fathers’ presence in the home, preschool, and out-of-home child care attendance, psychometrician, location, and language of assessment) confounders. Tier 1 and Tier 2 confounders were consistent with the previously completed systematic review [23]. For all tools, we decided that studies including all Tier 1 *and* Tier 2 confounders were rated as low ROB. Studies including all Tier 1 confounders were rated as probably low ROB. Studies including only some Tier 1 confounders or reported only crude analyses were rated as probably high and high ROB, respectively.

After completing the training, the two reviewers began by applying the OHAT tool to independently rate ROB for one article [31]. Studies included in the previous systematic review [23] were reviewed in alphabetical order by last name of the first author. The two reviewers reviewed the first study and then met, compared ratings, discussed discrepancies, came to consensus on ratings, and standardized their approach to improve the clarity of the OHAT instructions and maximize consistency in subsequent ratings. Researchers entered their ROB ratings and justification in Microsoft Excel. After assessing ROB using the OHAT tool in the remaining studies, the two reviewers met to discuss their ratings. In the event that the reviewers had different ratings, a consensus was reached via discussion. A third reviewer (JL) was brought in to resolve any discrepancies in case consensus could not be reached. This same approach was also used for applying first the IRIS then the TSCA tool.

Each reviewer tracked the time it took to complete the ROB assessment for each tool. The time to apply each tool has been used in previous study as an indicator of ease of use for ROB tools [14]. The total time and average time to review individual studies was calculated for the individual tools. We standardized this estimate by dividing by the number of questions on each tool. Kappa statistics were calculated for each tool as measures of inter-rater reliability using the package “IRR” in R version 3.6.0. Kappa values range from 0 to 100%, where 0% indicates no agreement and 100% indicates perfect agreement.

## Results

A description of the different tools, domains assessed, and number of questions and available answer options for each tool is provided in Table 1 and a detailed description of what each domain measures is provided in the Supplemental Information (Table S1).

It took approximately 5 h for each reviewer to review all 15 studies using the OHAT tool (average 20 min per study and 3 min per question); for the IRIS tool, it was approximately 8 h (average of 32 min per study and 4 min per question); and the TSCA tool was the longest, approximately 10 h (average 40 min per study and 2 min per question). Kappa values ranged from 54 to 58%.

### Similarities and differences across tools

**Fig. 1:**
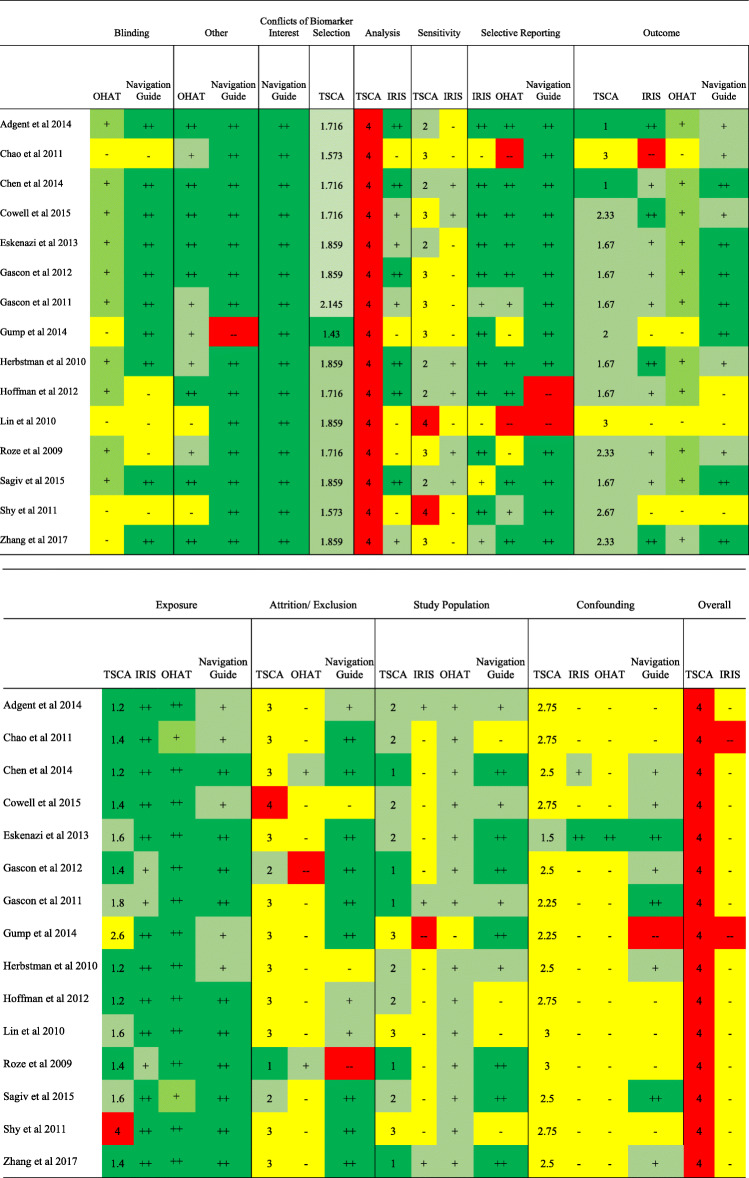
Comparison of risk of bias determinations for Navigation Guide, OHAT, IRIS, and TSCA tools. Abbreviations: OHAT, Office of Health Assessment and Translation; IRIS, Integrated Risk Information System; TSCA, Toxic Substances Control Act; ROB, risk of bias. Note: Double plus sign indicates low ROB, single plus sign indicates probably low ROB, single minus sign indicates probably high ROB, double minus sign indicates high ROB for IRIS, OHAT, and Navigation Guide. 1 indicates high, 2 indicates medium, 3 indicates low, 4 indicates unacceptable for TSCA. Study population for TSCA pertains to question 1. Attrition/exclusion for TSCA pertains to question 2. Sensitivity for TSCA pertains to question 5. Scores for TSCA were calculated by using the weighted sum of the individual questions within each domain

We found consistent ratings across the Navigation Guide, OHAT, IRIS, and TSCA tools for the domains of exposure characterization, outcome measurement, and confounding (Fig. 1). We found ratings of low or probably low bias across the tools for exposure characterization (Fig. 1). For outcome measurement, ratings were also consistent with the same four studies rated down across the tools in this domain [32–35]. Nearly all studies were considered to be at a high ROB due to confounding when using the IRIS, OHAT, and TSCA tools (Fig. 1).

We found differences in ratings across the tools for the domains of selection of study participants and selective reporting (Fig. 1). The Navigation Guide, OHAT, and TSCA tools generally rated studies as having low ROB due to selection of study participants. In contrast, the majority of studies were rated as being of medium or high ROB due to selection of study participants using IRIS. For selective reporting, using the IRIS tool, 12 out of 15 studies received “good” or “adequate” ratings for this domain. A different set of 2 studies received a rating of “probably high” ROB using OHAT [32, 36], these studies were generally not rated as having a high ROB in the other tools. We rated an additional 2 out of the 15 studies as “definitely high” ROB due to selective reporting using the OHAT tool [33, 34], these studies received similar ROB ratings for this question using the other tools. One of these studies was classified as “high” ROB using the Navigation Guide, along with one other study [37]. A detailed description of the final consensus decisions for each individual study across the IRIS, TSCA, and OHAT tools is provided in the Supplemental Information (Tables S3-S17).

Differences were also observed between the tools with respect to overall study confidence, as the IRIS and TSCA tools were the only ones to calculate overall study confidence metrics. All studies were retained in the body of evidence using the OHAT and Navigation Guide methods. In contrast, the IRIS and TSCA tools substantially reduced the number of studies available.

### Overall study confidence determinations using OHAT

**Fig. 2:**
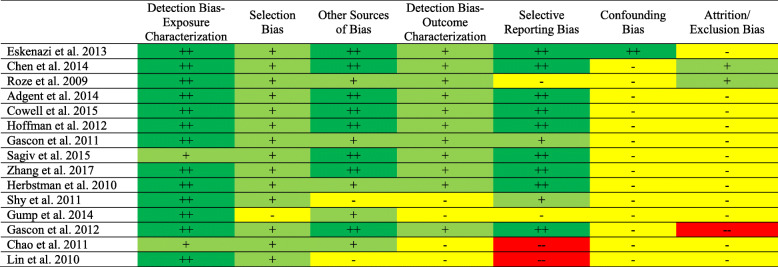
Summary of risk of bias judgments (low, probably low, probably high, high) using the OHAT framework for the human studies included in our case series. The justification for risk of bias designations for individual studies are provided in Tables S3-S17. Kappa value was 56% (95% confidence interval 44-66%). Note: Double plus sign indicates low, single plus sign indicates probably low, single minus sign indicates probably high, double minus sign indicates high study quality

When evaluating the confidence of the body of evidence using the OHAT tool, approximately half of the studies had “low” or “probably low” ROB ratings across all domains, with the exception of confounding bias and attrition/exclusion bias. Seven out of 15 studies received “probably high” or “high” ratings across three or more questions (Fig. 2).

### Overall study confidence determinations using IRIS

**Fig. 3:**
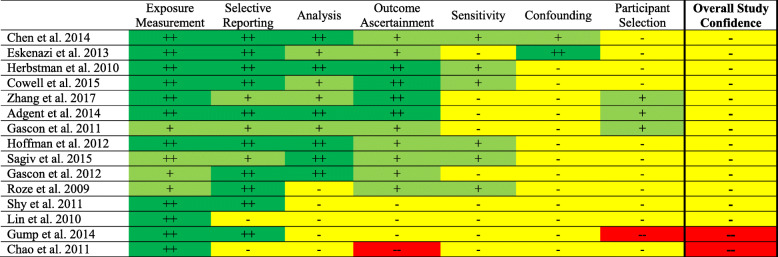
Summary of risk of bias judgments (good, adequate, deficient, critically deficient) using the IRIS framework for the human studies included in our case series. The justification for risk of bias designations for individual studies are provided in Tables S3-S17. Kappa value was 58% (95% confidence interval 48-69%). Note: For individual domains double plus sign indicates good, single plus sign indicates adequate, single minus sign indicates deficient, and double minus sign indicates critically deficient. For overall study confidence double plus sign indicates high, single plus sign indicates medium, single minus sign indicates low, and double minus sign indicates uninformative study quality

Using the IRIS tool, every study was rated either “low” or “uninformative” overall study confidence, with 13 studies rated “low” and two studies rated “uninformative” (Fig. 3). Most studies received “low” overall ratings as a result of bias due to confounding, participant selection, or sensitivity.

The sensitivity analysis examined a different definition for overall study confidence using the IRIS tool, which led to different overall study ratings. One study was rated as “high,” nine studies were rated as “medium,” and five studies were determined to have “low” overall study confidence, respectively (see Supplemental Information Figure S2). Consistent with our main findings, study confidence was generally downgraded from “high” or “medium” to “low” confidence as a result of bias due to analysis, confounding, and/or participant selection.

### Overall study confidence determinations using TSCA

**Fig. 4:**
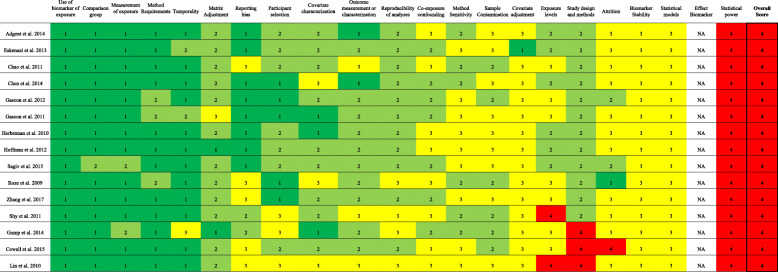
Summary of risk of bias judgments (high, medium, low, unacceptable) using the TSCA framework for the human studies included in our case series. The justification for risk of bias designations for individual studies are provided in Tables S3-S17. Kappa value was 54% (95% confidence interval 47-61%). Note: 1 indicates high, 2 indicates medium, 3 indicates low, 4 indicates unacceptable study quality. Abbreviations: NA, not applicable

All studies were rated “unacceptable” for overall study quality using the TSCA tool (Fig. 4). This occurred as a result of all studies being rated “unacceptable” for the question pertaining to statistical power. For the statistical power question, answer options included “medium” and “unacceptable” only (Table 1). To be rated “medium,” the number of participants needed to be adequate to detect an effect in the exposed population or study authors had to report that they had ≥ 80% power. No studies reported a power calculation and most studies had relatively small sample sizes.

Two studies [34, 35] were additionally rated “unacceptable” for bias due to exposure levels and three others [32, 34, 38] for bias due to study design and methods using TSCA. For the exposure levels question, answer options were “medium,” “low,” and “unacceptable” only. To be rated “unacceptable,” reviewers determined that the distribution of exposure was not adequate to determine an exposure-outcome relationship or study authors did not provide information on the range of exposure levels. Answer options for the study design and methods question were “medium” and “unacceptable” only. Studies were rated “unacceptable” for this question if inappropriate statistical analyses were applied or if the study design was not appropriate for the research question.

### ROB determinations using the Navigation Guide

Final ROB determinations made using the Navigation Guide in the original review are shown in the Supplemental Information (Figure S1) and final consensus decisions for the Navigation Guide is provided elsewhere [23]. Briefly, most studies received ratings of “low” or “probably low” ROB across most domains. All studies were free of conflicts of interest and all studies were rated as “low” or “probably low” ROB due to exposure assessment. With the exception of two studies, all studies were rated “low” ROB due to selective reporting [34, 37]. Approximately half of studies were rated as “high” or “probably high” ROB due to confounding [31–37] and a third of studies were rated as “probably high” ROB due to blinding [33–37].

## Discussion

The goal of our study was to compare three tools, OHAT, IRIS, and TSCA, for assessing ROB in a case study of human environmental observational evidence of PBDEs and IQ and/or ADHD. We also compared these tools to the Navigation Guide. Our results find that the use of these different tools can lead to different conclusions about the overall body of evidence, which has important implications for regulation of hazardous chemicals and public health. When the IRIS tool was applied, all studies were determined to have “low” or “uninformative” confidence in the overall study quality. Similarly, using the TSCA tool, all studies were determined to be of “uninformative” study quality, with no evidence of sufficient quality to be used to formulate an overall conclusion. In contrast, the OHAT tool did not include an overall quality indicator for individual studies, which allowed all studies to be retained in the body of evidence.

Our findings are consistent with other studies that have assessed systematic review methods, namely that using different tools can result in different conclusions [15] and that single ROB summary scores can be misleading [39]. All ROB tools assessed here showed overlap in some of the domains assessed, which supports previous findings [39]. For example, there is some consistency in the consideration of the domains across the Navigation Guide, IRIS, and OHAT tools for how they evaluate confounding, exposure assessment, outcome assessment, and selective reporting as they have been derived from the domains with empirical testing in randomized clinical trials. Further, as these domains are derived from the empirical tests of randomized clinical trials, they minimize over or double counting bias domains.

However, an important distinction between the IRIS, OHAT, and TSCA tools is that the IRIS tool includes a subjective indicator, as opposed to a weighted average or similar, for overall study quality. In our main analysis, all studies were downgraded when assessing ROB and the overall study quality due to “low” or “uninformative” overall study confidence determinations. These ratings were consistent with the guidance provided in the IRIS handbook. However, there is a great deal of flexibility in how overall study confidence is determined with the IRIS tool. For example, the IRIS tool allows studies to be classified as “medium” study confidence if there is a deficient rating in a domain that is considered to have less influence on the direction of the effect estimate. However, the handbook does not define which domains have less influence and we were unable to find scientific evidence to support judgments of certain domains as being more influential than others. We felt that rating a study as “medium” or “low” overall study confidence based on measures that have not been validated or empirically demonstrated was concerning, as it may result in the exclusion of studies that are ultimately informative when assessing the harms of hazardous exposures. Thus, in our sensitivity analyses, we used a more lenient criterion for assessing overall study confidence. For the sensitivity analysis, we found that only five studies (33%) were removed from the overall quality of evidence due to “low” overall study confidence determinations. It is possible that other reviewers would have vastly different interpretations of the overall study confidence question, which could lead to substantial differences in the number of studies retained in the overall body of evidence, as we have demonstrated.

All studies were considered to be “uninformative” using the TSCA tool, as all studies were rated “unacceptable ROB” for the question regarding statistical power. This resulted in the exclusion of all studies from the body of evidence. Notably, most studies included in our case study were prospective cohort studies, which are considered a strong evidence base for environmental health, and should lead to increased confidence in the rating as a result of better control for confounders as opposed to cross-sectional or case-control studies. For the statistical power question, the available answer options were limited to only “medium” (indicative of a probably low ROB) and “unacceptable” (indicative of a high ROB). To be considered “medium,” the study had to report ≥ 80% power to detect an effect or that the number of exposed participants was adequate to detect an effect. However, it is suggested that it is inappropriate to conduct post hoc power calculations once statistical analysis is complete and so even if a study had ≥ 80% power, if it did not report the results from a power calculation it was to be rated “unacceptable” [40]. The wording of the TSCA tool’s statistical analysis question essentially ensures that all epidemiologic studies are rated “unacceptable” and effectively removes epidemiologic studies from the body of evidence unless there are large, statistically significant findings for all exposures of interest. Importantly, sample size is one contributing factor to statistical power which may influence precision [6]. Small studies often produce imprecise effect estimates, which does not necessarily mean that they are biased. As demonstrated in the previously completed systematic review we used to evaluate these tools [23], small studies can demonstrate a statistically significant and precise association between exposure and outcomes when combined in a meta-analysis that increases the statistical power of the body of evidence.

An additional limitation of the TSCA tool is the broad domains comprised of 2-7 individual questions. When applying the tool, the individual questions for each domain appear to be very similar and it was often hard for study reviewers to differentiate between them. For example, domain 4 covers potential confounding/variable control and includes one question for covariate adjustment, one question for covariate characterization, and one question for co-exposure confounding. When reading these questions, the reviewers applying the TSCA tool found it difficult to separate out the ratings for each of these questions so that underlying limitations would not be represented more than once. We found this troubling, as other reviewers may have rated the evidence differently and experienced more difficulties. Therefore, studies could be downgraded or excluded multiple times from the overall body of evidence due to one aspect of the study design.

Although both the IRIS and TSCA tools include an indicator for overall study confidence, there exist key differences in how this indicator rating is determined. The IRIS tool weights each domain equally and reviewers determine the overall rating based on considering the ratings across several domains. In contrast, the TSCA tool uses a numerical scoring system which weights individual questions. Despite using different indicators for overall study quality, we found that both the IRIS and TSCA tools excluded all studies from further evaluation based on the rating from one or two domains or metrics. Importantly, by using these overall scoring systems, it is impossible to determine the differences between studies that were rated down for a single question or multiple questions [14]. Furthermore, there is no empirical evidence demonstrating how each ROB domain should be weighted [41] and the exclusion of studies based on an arbitrary rating of the evidence is not supported. It has also been empirically demonstrated overall “quality scores” are unable to distinguish between studies with a high or low ROB in meta-analyses [20, 42]. Thus, including only “high” quality studies may lead to a biased evaluation of the evidence, as there is no scientific justification for the use of overall quality scoring measures. If studies are to be excluded from a body of evidence, it is more appropriate to evaluate their influence on the overall effect estimates quantitatively using meta-analysis. Strategies including conducting sensitivity analyses which calculate overall effect estimates among high quality studies only or stratifying results based on overall study quality. Researchers may also choose to present all studies and qualitatively discuss the ROB using structured approaches, similar to OHAT and GRADE [43].

The TSCA tool also includes study reporting measures in its scoring of studies. Study reporting addresses how well a study’s findings are detailed, which contrasts with ROB which is designed to assess internal validity of a study and research quality. Within the TSCA tool, these study reporting guidelines are incorporated into the justification for rating studies as “low” (metrics 1 and 15) or “unacceptable” (metrics 2-7). Validated guidelines and checklists to enhance study reporting already exist (see Strengthening of Reporting of Observational Studies in Epidemiology [STROBE]) [44]. These guidelines have been developed to help ensure authors present all information needed to assess the quality and meaning of the research in the study. Importantly, STROBE guidelines specifically state that indicators of study reporting are not a measure of the quality of the underlying research [44].

An important goal of our study was to assess the ease and feasibility of using the different tools. The kappa statistics indicated moderate inter-rater reliability for all the different tools and providing more detail for each individual question could help to improve the agreement across all of the tools assessed here. Nonetheless, the inter-rater reliability findings are consistent with previous research assessing the use of different ROB tools that also found moderate kappa values when using the ROBINS-I (of which the IRIS tool is adapted) tool [45].

Our study has a number of important strengths. To the best of our knowledge, this is the first time that the reliability and validity of these different tools has been tested and compared. Additionally, we had two reproductive epidemiologists and a team member with expertise in systematic review and evaluating ROB complete the ROB assessments.

We also acknowledge our limitations. Our team did not include a neurodevelopment or biomarker assessment subject matter expert, and inclusion of these individuals may have led to different ratings for some of the domains. For example, in our case study, we assessed confounding using a yes/no approach where the study had to either adjust for all confounders on our pre-determined list or report that the confounders were evaluated and omitted because they did not influence the results. When the Navigation Guide tool was applied in the previously completed systematic review, expert judgment was applied in determining if including additional confounders would change the study results, which was outlined in their protocol [23]. It is possible that inclusion of additional subject matter experts on our research team would have led to different conclusions for the confounding domains within these different tools. However, we note that the transparency of systematic review ensures that the justification for conclusions is readily available for an independent reviewer to identify where expert judgment was applied and where one might disagree with this judgment. Additionally, we did not randomize the order of the studies or the tools, which may have influenced the average time it took to review the studies using each of the tools, resulting from increasing familiarity with the studies as the reviewers applied each tool sequentially. However, the average time to increase studies increased across the tools as they were applied, thus indicating that the time to apply the IRIS and TSCA tools would likely be higher if applied to a study being evaluated for the first time. Lastly, our case study was based on a relatively small number of studies (*n* = 15) and we may have more confidence in our results if our results are consistent upon replication in a larger number of studies. Additionally, it is possible that the kappa values observed in our study would have been higher if a larger number of studies were included.

### Conclusions

Systematic reviews are becoming increasingly important in environmental health and our case study finds that using these different tools can lead to opposite conclusions regarding the body of evidence. Tools that use an overall ROB or study quality rating based on weighting of domains or scoring metrics that have not been validated or empirically demonstrated may lead to erroneous conclusions about the quality of the body of evidence, as these tools may only consider a subset of studies when drawing conclusions. Further, the exclusion of studies based off only one “unacceptable” or “critically deficient” criterion can significantly reduce the available evidence to assess the harms of hazardous environmental exposures, which could lead to underestimating the health effects of hazardous chemicals and thus inadequate support for regulation of hazardous chemicals. When assessing ROB in systematic reviews, we recommend using tools that use validated, domain based approaches which do not exclude studies based off one single criterion. Rather, tools should consider the strengths and limitations of the entire body of evidence when formulating conclusions. Examples of these methods include the OHAT or Navigation Guide.

## Supplementary information

**Additional file 1: Table S1.** Description of domains measured across tools. **Figure S1.** Summary of risk of bias judgments (low, probably low, probably high, high) using the Navigation Guide framework for the human studies included in our case series. Risk of bias designations for individual studies and the justification for each study is provided in Lam et al. Note: ++ indicates low, + indicates probably low, - indicates probably high, -- indicates high. **Figure S2.** Results from sensitivity analysis of risk of bias judgments (good, adequate, deficient, critically deficient) using the IRIS framework for the human studies included in our case series. The justification for risk of bias designations for individual studies are provided in Tables S2-S16. Note: ++ indicates good, + indicates adequate, - indicates deficient, -- indicates critically deficient. Instructions for making risk of bias determinations using OHAT framework. Instructions for making risk of bias determinations using TSCA framework. **Table S2.** Metric Weighting Factors and Range of Weighted Metric Scores for Scoring the Quality of Epidemiology Studies. **Table S3.** Risk of bias ratings using the Adgent et al. (2014) study **Table S4.** Risk of bias ratings using the Chao et al. (2011) study **Table S5.** Risk of bias ratings using the Chen et al. (2014) study **Table S6.** Risk of bias ratings using the Cowell et al. (2015) study **Table S7.** Risk of bias ratings using the Eskenazi et al. (2013) study **Table S8.** Risk of bias ratings using the Gascon et al. (2012) study **Table S9.** Risk of bias ratings using the Gascon et al. (2011) study **Table S10.** Risk of bias ratings using the Gump et al. (2014) study **Table S11.** Risk of bias ratings using the Herbstman et al. (2010) study **Table S12.** Risk of bias ratings using the Hoffman et al. (2012) study **Table S13.** Risk of bias ratings using the Lin et al. (2010) study **Table S14.** Risk of bias ratings using the Roze et al. (2009) study **Table S15.** Risk of bias ratings using the Sagiv et al. (2015) study **Table S16.** Risk of bias ratings using the Shy et al. (2011) study **Table S17.** Risk of bias ratings using the Zhang et al. (2017) study

## Data Availability

All data generated or analyzed during this study are included in this published article and its supplementary information files.

## Abbreviations

OHAT: Office of Health Assessment and Translation
IRIS: Integrated Risk Information System
TSCA: Toxic Substances Control Act
NTP: National Toxicology Program
NAS: National Academy of Sciences
UCSF: University of California, San Francisco
EPA: Environmental Protection Agency
ROB: Risk of bias
PBDE: Polybrominated diphenyl ethers
IQ: Intelligence quotient
ADHD: Attention deficit hyperactivity disorder
