# Supplementary material for: Assessing risk of bias in human environmental epidemiology studies using three tools: different conclusions from different tools
Source: Syst Rev. 2020 Oct 29;9:249. doi: 10.1186/s13643-020-01490-8 (PMC7596989; doi:10.1186/s13643-020-01490-8)
Supplement: Supplementary file 1 — Additional file 1: Table S1. Description of domains measured across tools. Figure S1. Summary of risk of bias judgments (low, probably low, probably high, high) using the Navigation Guide framework for the human studies included in our case series. Risk of bias designations for individual studies and the justification for each study is provided in Lam et al. Note: ++ indicates low, + indicates probably low, - indicates probably high, -- indicates high. Figure S2. Results from sensitivity analysis of risk of bias judgments (good, adequate, deficient, critically deficient) using the IRIS framework for the human studies included in our case series. The justification for risk of bias designations for individual studies are provided in Tables S2-S16. Note: ++ indicates good, + indicates adequate, - indicates deficient, -- indicates critically deficient. Instructions for making risk of bias determinations using OHAT framework. Instructions for making risk of bias determinations using TSCA framework. Table S2. Metric Weighting Factors and Range of Weighted Metric Scores for Scoring the Quality of Epidemiology Studies. Table S3. Risk of bias ratings using the Adgent et al. (2014) study Table S4. Risk of bias ratings using the Chao et al. (2011) study Table S5. Risk of bias ratings using the Chen et al. (2014) study Table S6. Risk of bias ratings using the Cowell et al. (2015) study Table S7. Risk of bias ratings using the Eskenazi et al. (2013) study Table S8. Risk of bias ratings using the Gascon et al. (2012) study Table S9. Risk of bias ratings using the Gascon et al. (2011) study Table S10. Risk of bias ratings using the Gump et al. (2014) study Table S11. Risk of bias ratings using the Herbstman et al. (2010) study Table S12. Risk of bias ratings using the Hoffman et al. (2012) study Table S13. Risk of bias ratings using the Lin et al. (2010) study Table S14. Risk of bias ratings using the Roze et al. (2009) study Table S15. Risk of bias ratings using the Sagiv et al. [file 13643_2020_1490_MOESM1_ESM.docx]

Table S1. Description of domains measured across tools.

| **Tool** | **Domain** | **Description** |
| --- | --- | --- |
| OHAT | Selection Bias | Selection of study participants |
|  | Confounding Bias | Confounding |
|  | Attrition/Exclusion Bias | Incomplete outcome data |
|  | Detection Bias | Exposure and outcome characterization |
|  | Selective Bias Reporting Bias | Reporting of all analyses and outcomes described |
|  | Other Bias | Other threats to internal validity |
| IRIS | Exposure Measurement | Exposure characterization |
|  | Outcome Ascertainment | Outcome characterization |
|  | Participant Selection | Selection of study participants |
|  | Confounding | Confounding |
|  | Analysis | Appropriateness of statistical methods |
|  | Selective Reporting | Reporting of all analyses described |
|  | Sensitivity | Levels of exposure |
| TSCA | Study Population | Selection of study participants, missing data, appropriateness of comparison group |
|  | Exposure Characterization | Exposure characterization, levels of exposure, temporality |
|  | Outcome Assessment | Outcome characterization, reporting of all outcomes described |
|  | Potential Confounding/Variable Control | Covariate adjustment, covariate measurement, co-exposures |
|  | Analysis | Study design, statistical power, reproducibility, appropriateness of statistical methods |
|  | Biomarker Selection and Measurement | Accuracy of biomarker, sensitivity of biomarker, stability of biomarker, possible contamination of sample used to measure biomarker, method used to measure biomarker |
| Navigation Guide | Source Population Representation | Selection of study participants |
|  | Blinding | Exposure and outcome group assessments were blinded/masked |
|  | Exposure Assessment | Exposure characterization |
|  | Outcome Assessment | Outcome characterization |
|  | Incomplete Outcome Data | Incomplete outcome data |
|  | Selective Outcome Reporting | Reporting of all outcomes and analyses described |
|  | Confounding | Confounding |
|  | Conflicts of Interest | Support from a company, study author, or other having a financial interest in any the exposures studied |
|  | Other | Other |

|  | Source Population Representation | Blinding | Exposure Assessment | Outcome Assessment | Incomplete Outcome Data | Selective Outcome Reporting | Confounding | Conflicts of Interest | Other Sources of Bias |
| --- | --- | --- | --- | --- | --- | --- | --- | --- | --- |
| Adgent et al. 2014 | + | ++ | + | + | + | ++ | - | ++ | ++ |
| Chao et al. 2011 | - | - | + | + | ++ | ++ | - | ++ | ++ |
| Chen et al. 2014 | ++ | ++ | ++ | ++ | ++ | ++ | + | ++ | ++ |
| Cowell et al. 2015 | + | ++ | + | + | - | ++ | + | ++ | ++ |
| Eskenazi et al. 2013 | ++ | ++ | ++ | ++ | ++ | ++ | ++ | ++ | ++ |
| Gascon et al. 2012 | ++ | ++ | ++ | ++ | ++ | ++ | + | ++ | ++ |
| Gascon et al. 2011 | + | ++ | ++ | ++ | ++ | ++ | ++ | ++ | ++ |
| Gump et al. 2014 | ++ | ++ | + | ++ | ++ | ++ | -- | ++ | -- |
| Herbstman et al. 2010 | + | ++ | + | + | - | ++ | + | ++ | ++ |
| Hoffman et al. 2012 | - | - | ++ | - | + | -- | - | ++ | ++ |
| Lin et al. 2010 | - | - | ++ | - | + | -- | - | ++ | ++ |
| Roze et al. 2009 | ++ | - | ++ | + | -- | ++ | - | ++ | ++ |
| Sagiv et al. 2015 | ++ | ++ | ++ | ++ | ++ | ++ | ++ | ++ | ++ |
| Shy et al. 2011 | - | - | ++ | - | ++ | ++ | - | ++ | ++ |
| Zhang et al. 2017 | ++ | ++ | ++ | ++ | ++ | ++ | + | ++ | ++ |

**Figure S1. Summary of risk of bias judgments (low, probably low, probably high, high) using the Navigation Guide framework for the human studies included in our case series. Risk of bias designations for individual studies and the justification for each study is provided in Lam et al.**

Note: ++ indicates low, + indicates probably low, - indicates probably high, -- indicates high.

|  | Exposure Measurement | Selective Reporting | Analysis | Outcome Ascertainment | Sensitivity | Confounding | Participant Selection | **Overall Study Confidence** |
| --- | --- | --- | --- | --- | --- | --- | --- | --- |
| Chen et al. 2014 | ++ | ++ | ++ | + | + | + | - | **++** |
| Eskenazi et al. 2013 | ++ | ++ | + | + | - | ++ | - | **+** |
| Herbstman et al. 2010 | ++ | ++ | ++ | ++ | + | - | - | **+** |
| Cowell et al. 2015 | ++ | ++ | + | ++ | + | - | - | **+** |
| Zhang et al. 2017 | ++ | + | + | ++ | - | - | + | **+** |
| Adgent et al. 2014 | ++ | + | + | ++ | - | - | + | **+** |
| Gascon et al. 2011 | + | + | + | + | - | - | + | **+** |
| Hoffman et al. 2012 | ++ | ++ | ++ | + | + | - | - | **+** |
| Sagiv et al. 2015 | ++ | + | ++ | + | + | - | - | **+** |
| Gascon et al. 2012 | + | ++ | ++ | + | - | - | - | **+** |
| Roze et al. 2009 | + | ++ | - | + | + | - | - | **-** |
| Shy et al. 2011 | ++ | ++ | - | - | - | - | - | **-** |
| Lin et al. 2010 | ++ | - | - | - | - | - | - | **-** |
| Gump et al. 2014 | ++ | ++ | - | - | - | - | -- | **-** |
| Chao et al. 2011 | ++ | - | - | -- | - | - | - | **-** |

**Figure S2. Results from sensitivity analysis of risk of bias judgments (good, adequate, deficient, critically deficient) using the IRIS framework for the human studies included in our case series. The justification for risk of bias designations for individual studies are provided in Tables S2-S16.**

Note: ++ indicates good, + indicates adequate, - indicates deficient, -- indicates critically deficient.

**Instructions for making risk of bias determinations using OHAT framework.**

Please answer “Definitely low risk of bias”, “Probably low risk of bias”, “Probably high risk of bias”, and “Definitely high risk of bias”.

1. **Selection Bias: Did selection of study participants result in appropriate comparison groups?**
   1. Definitely low risk of bias: There is direct evidence that subjects (both exposed and non-exposed) were similar (e.g., recruited from the same eligible population, recruited with the same method of ascertainment using the same inclusion and exclusion criteria, and were of similar age and health status), recruited within the same time frame, and had the similar participation/response rates.
   2. Probably low risk of bias: There is indirect evidence that subjects (both exposed and non-exposed) were similar (e.g., recruited from the same eligible population, recruited with the same method of ascertainment using the same inclusion and exclusion criteria, and were of similar age and health status), recruited within the same time frame, and had the similar participation/response rates, **OR** differences between groups would not appreciably bias results.
   3. Probably high risk of bias: There is indirect evidence that subjects (both exposed and non-exposed) were not similar, recruited within very different time frames, or had the very different participation/response rates, there is insufficient information provided about the comparison group including a different rate of non-response without an explanation (record “NR” as basis for answer).
   4. Definitely high risk of bias: There is direct evidence that subjects (both exposed and non-exposed) were not similar, recruited within very different time frames, or had the very different participation/response rates.
2. **Confounding Bias: Did the study design or analysis account for important confounding and modifying variables?**
   1. **Definitely Low risk of bias:** There is direct evidence that appropriate adjustments or explicit considerations were made for primary covariates and confounders in the final analyses through the use of statistical models to reduce research-specific bias including standardization, matching, adjustment in multivariate model, stratification, propensity scoring, or other methods that were appropriately justified. Acceptable consideration of appropriate adjustment factors includes cases when the factor is not included in the final adjustment model because the author conducted analyses that indicated it did not need to be included, **AND** there is direct evidence that primary covariates and confounders were assessed using valid and reliable measurements, **AND** there is direct evidence that other exposures anticipated to bias results were not present or were appropriately measured and adjusted for. In occupational studies or studies of contaminated sites, other chemical exposures known to be associated with those settings were appropriately considered.
   2. **Probably Low risk of bias:** There is indirect evidence that appropriate adjustments were made, **OR** it is deemed that not considering or only considering a partial list of covariates or confounders in the final analyses would not appreciably bias results. **AND** there is evidence (direct or indirect) that primary covariates and confounders were assessed using valid and reliable measurements**, OR** it is deemed that the measures used would not appreciably bias results (i.e., the authors justified the validity of the measures from previously published research), **AND** there is evidence (direct or indirect) that other co-exposures anticipated to bias results were not present or were appropriately adjusted for, **OR** it is deemed that co-exposures present would not appreciably bias results.
   3. **Probably High risk of bias:** There is indirect evidence that the distribution of primary covariates and known confounders differed between the groups and was not appropriately adjusted for in the final analyses, **OR** there is insufficient information provided about the distribution of known confounders (record “NR” as basis for answer), **OR** there is indirect evidence that primary covariates and confounders were assessed using measurements of unknown validity, **OR** there is insufficient information provided about the measurement techniques used to assess primary covariates and confounders (record “NR” as basis for answer), **OR** there is indirect evidence that there was an unbalanced provision of additional co-exposures across the primary study groups, which were not appropriately adjusted for, **OR** there is insufficient information provided about co-exposures in occupational studies or studies of contaminated sites where high exposures to other chemical exposures would have been reasonably anticipated (record “NR” as basis for answer).
   4. **Definitely High risk of bias:** There is direct evidence that the distribution of primary covariates and known confounders differed between the groups, confounding was demonstrated, and was not appropriately adjusted for in the final analyses, **OR** there is direct evidence that primary covariates and confounders were assessed using non valid measurements, **OR** there is direct evidence that there was an unbalanced provision of additional co-exposures across the primary study groups, which were not appropriately adjusted for.
3. **Attrition/Exclusion Bias: Were outcome data complete without attrition or exclusion from analysis?**
   1. **Definitely Low risk of bias:** There is direct evidence that loss of subjects (i.e., incomplete outcome data) was adequately addressed and reasons were documented when human subjects were removed from a study. Acceptable handling of subject attrition includes: very little missing outcome data; reasons for missing subjects unlikely to be related to outcome (for survival data, censoring unlikely to be introducing bias); missing outcome data balanced in numbers across study groups, with similar reasons for missing data across groups, **OR** missing data have been imputed using appropriate methods and characteristics of subjects lost to follow up or with unavailable records are described in identical way and are not significantly different from those of the study participants.
   2. **Probably Low risk of bias:** There is indirect evidence that loss of subjects (i.e., incomplete outcome data) was adequately addressed and reasons were documented when human subjects were removed from a study, **OR** it is deemed that the proportion lost to follow-up would not appreciably bias results. This would include reports of no statistical differences in characteristics of subjects lost to follow up or with unavailable records from those of the study participants. Generally, the higher the ratio of participants with missing data to participants with events, the greater potential there is for bias. For studies with a long duration of follow-up, some withdrawals for such reasons are inevitable.
   3. **Probably High risk of bias:** There is indirect evidence that loss of subjects (i.e., incomplete outcome data) was unacceptably large and not adequately addressed, **OR** there is insufficient information provided about numbers of subjects lost to follow-up (record “NR” as basis for answer).
   4. **Definitely High risk of bias:** There is direct evidence that loss of subjects (i.e., incomplete outcome data) was unacceptably large and not adequately addressed. Unacceptable handling of subject attrition includes: reason for missing outcome data likely to be related to true outcome, with either imbalance in numbers or reasons for missing data across study groups; or potentially inappropriate application of imputation.
4. **Detection Bias Exposure Characterization: Can we be confident in the exposure characterization?**
   1. **Definitely Low risk of bias:** There is direct evidence that exposure was consistently assessed (i.e., under the same method and time-frame) using well-established methods that directly measure exposure (e.g., measurement of the chemical in air or measurement of the chemical in blood, plasma, urine, etc.), **OR** exposure was assessed using less-established methods that directly measure exposure and are validated against well-established methods.
   2. **Probably Low risk of bias:** There is indirect evidence that the exposure was consistently assessed using well-established methods that directly measure exposure, **OR** exposure was assessed using indirect measures (e.g., questionnaire or occupational exposure assessment by a certified industrial hygienist) that have been validated or empirically shown to be consistent with methods that directly measure exposure (i.e., inter-methods validation: one method vs. another).
   3. **Probably High risk of bias:** There is indirect evidence that the exposure was assessed using poorly validated methods that directly measure exposure, **OR** there is direct evidence that the exposure was assessed using indirect measures that have not been validated or empirically shown to be consistent with methods that directly measure exposure (e.g., a job-exposure matrix or self-report without validation) (record “NR” as basis for answer), **OR** there is insufficient information provided about the exposure assessment, including validity and reliability, but no evidence for concern about the method used (record “NR” as basis for answer).
   4. **Definitely High risk of bias:** There is direct evidence that the exposure was assessed using methods with poor validity, **OR** evidence of exposure misclassification (e.g., differential recall of self-reported exposure).
5. **Detection Bias Outcome Characterization: Can we be confident in the outcome assessment?**
   1. **Definitely Low risk of bias:** There is direct evidence that the outcome was assessed using well-established methods (e.g., the “gold standard” with validity and reliability >0.70 Genaidy *et al.* 2007), **AND** subjects had been followed for the same length of time in all study groups. Acceptable assessment methods will depend on the outcome, but examples of such methods may include: objectively measured with diagnostic methods, measured by trained interviewers, obtained from registries (Shamliyan *et al.* 2010), **AND** there is direct evidence that the outcome assessors (including study subjects, if outcomes were self-reported) were adequately blinded to the study group, and it is unlikely that they could have broken the blinding prior to reporting outcomes.
   2. **Probably Low risk of bias:** There is indirect evidence that the outcome was assessed using acceptable methods (i.e., deemed valid and reliable but not the gold standard) (e.g., validity and reliability ≥0.40 Genaidy *et al.* 2007), **AND** subjects had been followed for the same length of time in all study groups [Acceptable, but not ideal assessment methods will depend on the outcome, but examples of such methods may include proxy reporting of outcomes and mining of data collected for other purposes], **OR** it is deemed that the outcome assessment methods used would not appreciably bias results, **AND** there is indirect evidence that the outcome assessors (including study subjects, if outcomes were self-reported) were adequately blinded to the study group, and it is unlikely that they could have broken the blinding prior to reporting outcomes, **OR** it is deemed that lack of adequate blinding of outcome assessors would not appreciably bias results, which is more likely to apply to objective outcome measures.
   3. **Probably High risk of bias:** There is indirect evidence that the outcome assessment method is an insensitive instrument (e.g., a questionnaire used to assess outcomes with no information on validation), **OR** the length of follow up differed by study group, **OR** there is indirect evidence that it was possible for outcome assessors (including study subjects if outcomes were self-reported) to infer the study group prior to reporting outcomes, **OR** there is insufficient information provided about blinding of outcome assessors (record “NR” as basis for answer).
   4. **Definitely High risk of bias:** There is direct evidence that the outcome assessment method is an insensitive instrument, **OR** the length of follow up differed by study group, **OR** there is direct evidence for lack of adequate blinding of outcome assessors (including study subjects if outcomes were self-reported), including no blinding or incomplete blinding.
6. **Selective Reporting Bias: Were all measured outcomes reported?**
   1. **Definitely Low risk of bias:** There is direct evidence that all of the study’s measured outcomes (primary and secondary) outlined in the protocol, methods, abstract, and/or introduction (that are relevant for the evaluation) have been reported. This would include outcomes reported with sufficient detail to be included in meta-analysis or fully tabulated during data extraction and analyses had been planned in advance.
   2. **Probably Low risk of bias:** There is indirect evidence that all of the study’s measured outcomes (primary and secondary) outlined in the protocol, methods, abstract, and/or introduction (that are relevant for the evaluation) have been reported, **OR** analyses that had not been planned in advance (i.e., retrospective unplanned subgroup analyses) are clearly indicated as such and it is deemed that the unplanned analyses were appropriate and selective reporting would not appreciably bias results (e.g., appropriate analyses of an unexpected effect). This would include outcomes reported with insufficient detail such as only reporting that results were statistically significant (or not).
   3. **Probably High risk of bias:** There is indirect evidence that all of the study’s measured outcomes (primary and secondary) outlined in the protocol, methods, abstract, and/or introduction (that are relevant for the evaluation) have been reported, **OR** and there is indirect evidence that unplanned analyses were included that may appreciably bias results, **OR** there is insufficient information provided about selective outcome reporting (record “NR” as basis for answer).
   4. **Definitely High risk of bias:** There is direct evidence that all of the study’s measured outcomes (primary and secondary) outlined in the protocol, methods, abstract, and/or introduction (that are relevant for the evaluation) have not been reported. In addition to not reporting outcomes, this would include reporting outcomes based on composite score without individual outcome components or outcomes reported using measurements, analysis methods or subsets of the data (e.g., subscales) that were not pre-specified or reporting outcomes not pre-specified, or that unplanned analyses were included that would appreciably bias results.
7. **Other Bias: Were there no other potential threats to internal validity (e.g., statistical methods were appropriate and researchers adhered to the study protocol)**
   1. **Examples:**
      1. **Statistics:** Failure to statistically or experimentally adjust for litter in an animal study with a developmental outcome. The direction of the bias is away from the null towards a larger effect size (Haseman *et al.* 2001).
      2. **Deviations from the protocol:** Evidence of deviations in the protocol should be noted as direct (definitely high risk of bias) or indirect (probably high risk of bias). Given reporting practices it is unlikely that deviations from the protocol will be explicitly reported in most studies and therefore the bias is very difficult to assess. Caution should be taken so that studies that do provide a protocol and report deviations are not “punished” for having better reporting practices.

**Instructions for making risk of bias determinations using IRIS framework.**

Please answer “Good”, “Adequate”, “Deficient”, and “Critically deficient”.

1. **Exposure measurement: Does the exposure measure reliably distinguish between levels of exposure in a time window considered most relevant for a causal effect with respect to the development of the outcome?**
   1. **Good:** Valid exposure assessment methods used, which represent the etiologically relevant time period of interest. Exposure misclassification is expected to be minimal
   2. **Adequate:** Valid exposure assessment methods used, which represent the etiologically relevant time period of interest. Exposure misclassification may exist but is not expected to greatly change the effect estimate.
   3. **Deficient:** Valid exposure assessment methods used, which represent the etiologically relevant time period of interest. Specific knowledge about the exposure and outcome raise concerns about reverse causality, but there is uncertainty whether it is influencing the effect estimate. Exposed groups are expected to contain a notable proportion of unexposed or minimally exposed individuals, the method did not capture important temporal or spatial variation, or there is other evidence of exposure misclassification that would be expected to notably change the effect estimate.
   4. **Critically deficient:** Exposure measurement does not characterize the etiologically relevant time period of exposure or is not valid. There is evidence that reverse causality is very likely to account for the observed association. Exposure measurement was not independent of outcome status.
2. **Outcome ascertainment: Does the outcome measure reliably distinguish the presence or absence (or degree of severity) of the outcome?**
   1. **Good:** High certainty in the outcome definition (i.e., specificity and sensitivity), minimal concerns with respect to misclassification. Assessment instrument was validated in a population comparable to the one from which the study group was selected.
   2. **Adequate:** Moderate confidence that outcome definition was specific and sensitive, some uncertainty with respect to misclassification but not expected to greatly change the effect estimate. Assessment instrument was validated but not necessarily in a population comparable to the study group.
   3. **Deficient:** Outcome definition was not specific or sensitive. Uncertainty regarding validity of assessment instrument.
   4. **Critically deficient:** Invalid/insensitive marker of outcome. Outcome ascertainment is very likely to be affected by knowledge of, or presence of, exposure.
3. **Participant selection: Is there evidence that selection into or out of the study (or analysis sample) was jointly related to exposure and to outcome?**
   1. **Good:** Minimal concern for selection bias based on description of recruitment process (e.g., selection of comparison population, population-based random sample selection, recruitment from sampling frame including current and previous employees). Exclusion and inclusion criteria specified and would not induce bias. Participation rate is reported at all steps of study (e.g., initial enrollment, follow-up, selection into analysis sample). If rate is not high, there is appropriate rationale for why it is unlikely to be related to exposure (e.g., comparison between participants and nonparticipants or other available information indicates differential selection is not likely).
   2. **Adequate:** Enough of a description of the recruitment process to be comfortable that there is no serious risk of bias. Inclusion and exclusion criteria specified and would not induce bias. Participation rate is incompletely reported but available information indicates participation is unlikely to be related to exposure.
   3. **Deficient:** Little information on recruitment process, selection strategy, sampling framework and/or participation OR aspects of these processes raises the potential for bias (e.g., healthy worker effect, survivor bias).
   4. **Critically deficient:** Aspects of the processes for recruitment, selection strategy, sampling framework, or participation result in concern that selection bias is likely to have had a large impact on effect estimates (e.g., convenience sample with no information about recruitment and selection, cases and controls are recruited from different sources with different likelihood of exposure, recruitment materials stated outcome of interest and potential participants are aware of or are concerned about specific exposures)
4. **Confounding: Is confounding of the effect of the exposure likely?**
   1. **Good:** Conveys strategy for identifying key confounders. This may include: a priori biological considerations, published literature, causal diagrams, or statistical analyses; with recognition that not all “risk factors” are confounders. Inclusion of potential confounders in statistical models not based solely on statistical significance criteria (e.g., *p* < 0.05 from stepwise regression). Does not include variables in the models that are likely to be influential colliders or intermediates on the causal pathway. Key confounders are evaluated appropriately and considered to be unlikely sources of substantial confounding. This often will include: Presenting the distribution of potential confounders by levels of the exposure of interest and/or the outcomes of interest (with amount of missing data noted); Consideration that potential confounders were rare among the study population, or were expected to be poorly correlated with exposure of interest; Consideration of the most relevant functional forms of potential confounders; Examination of the potential impact of measurement error or missing data on confounder adjustment; Presenting a progression of model results with adjustments for different potential confounders, if warranted.
   2. **Adequate:** Similar to Good but may not have included all key confounders, or less detail may be available on the evaluation of confounders (e.g., sub-bullets in Good). It is possible that residual confounding could explain part of the observed effect, but concern is minimal.
   3. **Deficient:** Does not include variables in the models that are likely to be influential colliders or intermediates on the causal pathway. And any of the following: The potential for bias to explain some of the results is high based on an inability to rule out residual confounding, such as a lack of demonstration that key confounders of the exposure-outcome relationships were considered; Descriptive information on key confounders (e.g., their relationship relative to the outcomes and exposure levels) are not presented; or Strategy of evaluating confounding is unclear or is not recommended (e.g., only based on statistical significance criteria or stepwise regression [forward or backward elimination]);
   4. **Critically deficient:** Includes variables in the models that are colliders and/or intermediates in the causal pathway, indicating that substantial bias is likely from this adjustment; or Confounding is likely present and not accounted for, indicating that all of the results were most likely due to bias.
5. **Analysis: Does the analysis strategy and presentation convey the necessary familiarity with the data and assumptions?**
   1. **Good:** Use of an optimal characterization of the outcome variable. Quantitative results presented (effect estimates and confidence limits or variability in estimates) (i.e., not presented only as a *p*-value or “significant”/“not significant”). Descriptive information about outcome and exposure provided (where applicable). Amount of missing data noted and addressed appropriately (discussion of selection issues―missing at random vs. differential). Where applicable, for exposure, includes LOD (and percentage below the LOD), and decision to use log transformation. Includes analyses that address robustness of findings, e.g., examination of exposure-response (explicit consideration of nonlinear possibilities, quadratic, spline, or threshold/ceiling effects included, when feasible); relevant sensitivity analyses; effect modification examined based only on a priori rationale with sufficient numbers. No deficiencies in analysis evident. Discussion of some details may be absent (e.g., examination of outliers).
   2. **Adequate:** Same as Good, except: Descriptive information about exposure provided (where applicable), but may be incomplete; might not have discussed missing data, cutpoints, or shape of distribution. Includes analyses that address robustness of findings (examples in Good), but some important analyses are not performed.
   3. **Deficient:** Does not conduct analysis using optimal characterization of the outcome variable. Descriptive information about exposure levels not provided (where applicable). Effect estimate and *p*-value presented, without standard error or confidence interval. Results presented as statistically “significant”/“not significant.”
   4. **Critically deficient:** Results of analyses of effect modification examined without clear a priori rationale and without providing main/principal effects (e.g., presentation only of statistically significant interactions that were not hypothesis driven). Analysis methods are not appropriate for design or data of the study.
6. **Selective reporting: Is there reason to be concerned about selective reporting?**
   1. **Good:** The results reported by study authors are consistent with the primary and secondary analyses described in a registered protocol or methods paper.
   2. **Adequate:** The authors described their primary (and secondary) analyses in the methods section and results were reported for all primary analyses.
   3. **Deficient:** Concerns were raised based on previous publications, a methods paper, or a registered protocol indicating that analyses were planned or conducted that were not reported, or that hypotheses originally considered to be secondary were represented as primary in the reviewed paper. Only subgroup analyses were reported suggesting that results for the entire group were omitted. Only statistically significant results were reported.
7. **Sensitivity: Is there a concern that sensitivity of the study is not adequate to detect an effect?**
   1. **Adequate:** The range of exposure levels provides adequate variability to evaluate primary hypotheses in study. The population was exposed to levels expected to have an impact on response. The study population was sensitive to the development of the outcomes of interest (e.g., ages, life stage, sex). The timing of outcome ascertainment was appropriate given expected latency for outcome development (i.e., adequate follow-up interval). The study was adequately powered to observe an effect. No other concerns raised regarding study sensitivity.
   2. **Deficient:** Concerns were raised about the issues described for *good* that are expected to notably decrease the sensitivity of the study to detect associations for the outcome.
8. **Overall Study Confidence**
   1. **High:** All domains are good.
   2. **Medium:** Most domains are adequate or good.
   3. **Low:** Few domains are adequate or good.
   4. **Uninformative:** No domains are adequate or good.

**Instructions for making risk of bias determinations using TSCA framework.**

To meet criteria for confidence ratings for metrics where ‘AND’ is included, studies must address both conditions where “AND” is stipulated. To meet criteria for confidence ratings for metrics where ‘OR’ is included studies must address at least one of the conditions stipulated.

**Domain 1. Study Population**

1. **Participant selection (selection, performance biases):**
   1. **High (score=1):** All key elements of the study design are reported (e.g., setting, participation rate described at all steps of the study, inclusion and exclusion criteria, and methods of participant selection or case ascertainment) **AND** The reported information indicates that selection in or out of the study (or analysis sample) and participation was not likely to be biased (i.e., the exposure-outcome distribution of the participants is likely representative of the exposure-outcome distributions in the population of persons eligible for inclusion in the study.)
   2. **Medium (score=2):** Some key elements of the study design were not present but available information indicates a low risk of selection bias (i.e., the exposure-outcome distribution of the participants is likely representative of the exposure-outcome distributions in the population of persons eligible for inclusion in the study.)
   3. **Low (score=3):** Key elements of the study design and information on the population (e.g., setting, participation rate described at most steps of the study, inclusion and exclusion criteria, and methods of participant selection or case ascertainment) are not reported [STROBE checklist 4, 5 and 6 (Von Elm et al., 2008)].
   4. **Unacceptable (score=4):** The reported information indicates that selection in or out of the study (or analysis sample) and participation was likely to be significantly biased (i.e., the exposure-outcome distribution of the participants is likely not representative of the exposure-outcome distribution of the population of persons eligible for inclusion in the study.)
   5. **Not rated/ not applicable (NA):** Do not select for this metric.
2. **Attrition (missing data/attrition/exclusion, reporting biases)**
   1. **High (score=1):** There was minimal subject loss to follow up during the study (or exclusion from the analysis sample) and outcome and exposure data were largely complete. **OR** Any loss of subjects (i.e., incomplete outcome data) or missing exposure and outcome data were adequately* addressed (as described below) and reasons were documented when human subjects were removed from a study (NTP, 2015) **AND** Missing data have been imputed using appropriate methods (e.g., multiple imputation methods), and characteristics of subjects lost to follow up or with unavailable records are not significantly different from those of the study participants (NTP, 2015). ***** Adequate handling of subject attrition can include: Use of imputation methods for missing outcome and exposure data; reasons for missing subjects unlikely to be related to outcome (for survival data, censoring was unlikely to introduce bias); missing outcome data balanced in numbers across study groups, with similar reasons for missing data across groups.
   2. **Medium (score=2):** There was moderate subject loss to follow up during the study (or exclusion from the analysis sample) or outcome and exposure data were nearly complete. **AND** Any loss or exclusion of subjects was adequately addressed (as described in the acceptable handling of subject attrition in the high confidence category) and reasons were documented when human subjects were removed from a study.
   3. **Low (score=3):** The loss of subjects (e.g., loss to follow up, incomplete outcome or exposure data) was moderate and unacceptably handled (as described below in the unacceptable confidence category) (NTP, 2015) **OR** Numbers of individuals were not reported at important stages of study (e.g., numbers of eligible participants included in the study or analysis sample, completing follow-up, and analyzed). Reasons were not provided for nonparticipation at each stage (Von Elm et al., 2008).
   4. **Unacceptable (score=4): *For cohort studies:*** There was large subject attrition during the study (or exclusion from the analysis sample) **OR** Unacceptable handling of subject attrition: reason for missing outcome data likely to be related to true outcome, with either imbalance in numbers or reasons for missing data across study groups; or potentially inappropriate application of imputation (NTP, 2015).
   5. **Not rated/applicable:** Do not select for this metric.
3. **Comparison group (selection, performance biases)**
   1. **High (score=1):** Any differences in baseline characteristics of groups were considered as potential confounding or stratification variables and were thereby controlled by statistical analysis (NTP, 2015) **OR** Key elements of the study design are reported (i.e., setting, inclusion and exclusion criteria, and methods of participant selection), and indicate that subjects were similar (e.g., recruited from the same eligible population with the same method of ascertainment and within the same time frame using the same inclusion and exclusion criteria, and were of similar age and health status) (NTP, 2015).
   2. **Medium (score=2):** There is only indirect evidence (e.g., stated by the authors without providing a description of methods) that groups are similar (as described above for the high confidence rating).
   3. **Low (score = 3):** There is indirect evidence (i.e., stated by the authors without providing a description of methods) that groups were not similar (as described above for the high confidence rating) **AND** Control for differences in exposure groups is not adequately controlled for in the statistical analysis.
   4. **Unacceptable (score=4):** Subjects in all exposure groups were not similar **OR** Information was not reported to determine if participants in all exposure groups were similar [STROBE Checklist 6 (Von Elm et al., 2008) **AND** Potential differences in exposure groups were not controlled for in the statistical analysis. **OR** Subjects in the exposure groups had very different participation/response rates (NTP, 2015).
   5. **Not rated/applicable:** Do not select for this metric.

**Domain 2. Exposure Characterization**

1. **Measurement of Exposure (Detection/measurement/information, performance biases)**
   1. **High (score=1):** Exposure was consistently assessed (i.e., using the same method and sampling time-frame) using well-established methods (e.g., personal and/or industrial hygiene data used to determine levels of exposure, a frequently used biomarker of exposure) that directly measure exposure [e.g., measurement of the chemical in the environment (air, drinking water, consumer product] or measurement of the chemical concentration in a biological matrix (e.g., blood, plasma, urine) (NTP,2015) **OR** For an occupational population, contains detailed employment records which allows for construction of a job-matrix for entire work history of exposure (i.e., cumulative or peak exposures, and time since first exposure).
   2. **Medium (score=2):** Exposure was directly measured and assessed using a method that is not well-established (e.g., newly developed biomarker of exposure), ***but*** is validated against a well-established method and demonstrated a high agreement between the two methods **OR** for an occupational study population, contains detailed employment records for only a portion of participant’s work history. (i.e., only early years or later years), such that extrapolation of the missing years is required.
   3. **Low (score=3):** A less-established method (e.g., newly developed biomarker of exposure) was used and no method validation was conducted against well-established methods, but there was little to no evidence that the method had poor validity and little to no evidence of significant exposure misclassification (e.g., differential recall of self-reported exposure)(NTP, 2015).
   4. **Unacceptable (score=4):** Methods used to quantify the exposure were not well defined, and sources of data and detailed methods of exposure assessment were not reported [STROBE Checklist 7 and 8] **OR** Exposure was assessed using methods known or suspected to have poor validity (NTP, 2015) **OR** There is evidence of substantial exposure misclassification that would significantly bias the results.
   5. **Not rated/applicable:** Do not select for this metric.
2. **Exposure levels (Detection/measurement/information biases)**
   1. **High (score=1):** Do not select for this metric.
   2. **Medium (score=2):** The range and distribution of exposure is sufficient or adequate to develop an exposure-response estimate (Cooper et al., 2016) **AND** Reports 3 or more levels of exposure (i.e., referent group and 2 or more) or an exposure-response model using a continuous measure of exposure.
   3. **Low (score=3):** The range of exposure in the population is limited **OR** Reports 2 levels of exposure (e.g., exposed/unexposed)) (Cooper et al.,2016)
   4. **Unacceptable (score=4):** The range and distribution of exposure are not adequate to determine an exposure-response relationship (Cooper et al., 2016) **OR** No description is provided on the levels or range of exposure.
   5. **Not rated/applicable:** Do not select for this metric.
3. **Temporality (Detection/measurement/information biases)**
   1. **High (score=1):** The study presents an appropriate temporality between exposure and outcome (i.e. the exposure precedes the disease) **AND** The interval between the exposure (or reconstructed exposure) and the outcome has an appropriate consideration of relevant exposure windows (Lakind et al., 2014).
   2. **Medium (score=2):** Temporality is established, but it is unclear whether exposures fall within relevant exposure windows for the outcome of interest (Lakind et al., 2014).
   3. **Low (score=3):** The temporality of exposure and outcome is uncertain
   4. **Unacceptable (score=4): *For all study types:*** Study lacks an established time order, such that exposure is not likely to have occurred prior to outcome (Lakind et al., 2014) **OR** There was inadequate follow-up of the cohort for the expected latency period **OR** Sources of data and details of methods of assessment were not sufficiently reported (e.g. duration of follow-up, periods of exposure, dates of outcome ascertainment) [STROBE Checklist 8 (Von Elm et al., 2008)].
   5. **Not rated/applicable:** Do not select for this metric.

**Domain 3. Outcome Assessment**

1. **Outcome measurement or characterization (detection/measurement/information, performance, reporting biases)**
   1. **High (score=1):** The outcome was assessed using well-established methods (e.g., the “gold standard”).
   2. **Medium (score=2):** less-established method was used and no method validation was conducted against well-established methods, but there was little to no evidence that that the method had poor validity and little to no evidence of outcome misclassification (e.g., differential reporting of outcome by exposure status).
   3. **Low (score=3):** The outcome assessment method is an insensitive instrument or measure **OR** The length of follow up differed by study group (NTP, 2015).
   4. **Unacceptable (score=4):** Diagnostic criteria were not defined or reported [STROBE Checklist 15 (Von Elm et al., 2008)].
   5. **Not rated/applicable:** Do not select for this metric.
2. **Reporting Bias**
   1. **High (score=1):** description of measured outcomes is reported in the methods, abstract, and/or introduction. Effect estimates are reported with a confidence interval and/or standard errors; number of cases/controls or exposed/unexposed reported for each analysis, to be included in exposure response analysis or fully tabulated during data extraction and analyses (NTP, 2015).
   2. **Medium (score=2):** All of the study’s measured outcomes (primary and secondary) outlined in the methods, abstract, and/or introduction (that are relevant for the evaluation) are reported, but not in a way that would allow for detailed extraction (e.g., results were discussed in the text but accompanying data were not shown).
   3. **Low (score=3):** All of the study’s measured outcomes (primary and secondary) outlined in the methods, abstract, and/or introduction (that are relevant for the evaluation) have not been reported. ****Note:*** In addition to not reporting outcomes, this would include reporting outcomes based on composite score without individual outcome components or outcomes reported using measurements, analysis methods, or unplanned analyses were included that would appreciably bias results (NTP, 2015).
   4. **Unacceptable (score=4):** Do not select for this metric.
   5. **Not rated/applicable:** Do not select for this metric.

**Domain 4. Potential Confounding/Variable Control**

1. **Covariate Adjustment (confounding)**
   1. **High (score=1):** Appropriate adjustments or explicit considerations were made for potential confounders (e.g. age, sex, socioeconomic status) (excluding co-exposures, which are evaluated in metric 11) in the final analyses through the use of statistical models to reduce research-specific bias, including matching, adjustment in multivariate models, stratification, or other methods that were appropriately justified (NTP, 2015).
   2. **Medium (score=2):** There is indirect evidence that appropriate adjustments were made [i.e., considerations were made for potential confounders (excluding co-exposures)] without providing a description of methods **OR** The distribution of potential confounders (excluding co-exposures) did not differ significantly between exposure groups or between cases and controls **OR** The major potential confounders (excluding co-exposures) were appropriately adjusted (e.g., SMRs, SIRs) and any not adjusted for are considered not to appreciably bias the results
   3. **Low (score=3):** There is indirect evidence (i.e., no description is provided in the study) that considerations were not made for potential confounders adjustment in the final analyses (NTP, 2015) **AND** The distribution of primary covariates (excluding co-exposures) and potential confounders was not reported between the exposure groups or between cases and controls (NTP, 2015).
   4. **Unacceptable (score=4):** The distribution of potential confounders differed significantly between the exposure group **AND** Confounding was demonstrated and was not appropriately adjusted for in the final analyses (NTP, 2015).
   5. **Not rated/applicable:** Do not select for this metric.
2. **Covariate Characterization (measurement/information, confounding biases)**
   1. **High (score=1):** Potential confounders (excluding co-exposures; e.g. age, sex, SES) were assessed using valid and reliable methodology where appropriate (e.g., validated questionnaires, biomarker).
   2. **Medium (score=2):** less-established method was used to assess confounders (excluding co-exposures) and no method validation was conducted against well-established methods, but there was little to no evidence that that the method had poor validity and little to no evidence of confounding.
   3. **Low (score=3):** The confounder (excluding co-exposures) assessment method is an insensitive instrument or measure or a method of unknown validity.
   4. **Unacceptable (score=4):** Confounders were assessed using a method or instrument known to be invalid.
   5. **Not rated/applicable:** Covariates were not assessed.
3. **Co-exposure Confounding (measurement/information, confounding biases)**
   1. **High (score=1):** Do not select for this metric.
   2. **Medium (score=2):** Any co-exposures to pollutants that are not the target exposure that would likely bias the results were not likely to be present **OR** Co-exposures to pollutants were appropriately measured or either directly or indirectly adjusted for.
   3. **Low (score=3):** There is direct evidence that there was an unbalanced provision of additional co-exposures across the primary study groups, which were not appropriately adjusted for.
   4. **Unacceptable (score=4):** Do not select for this metric.
   5. **Not rated/applicable:** Enter ‘NA’ and do not score this metric.

**Domain 5. Analysis**

1. **Study Design and Methods**
   1. **High (score=1):** Do not select for this metric.
   2. **Medium (score=2):** The study design chosen was appropriate for the research question (e.g. assess the association between exposure levels and common chronic diseases over time with cohort studies, assess the association between exposure and rare diseases with case-control studies, and assess the association between exposure levels and acute disease with a cross-sectional study design) **AND**  The study uses an appropriate statistical method to address the research question(s) (e.g., repeated measures analysis for longitudinal studies, logistic regression analysis for case-control studies, or mean, median for descriptive studies)
   3. **Low (score=3):** Do not select for this metric.
   4. **Unacceptable (score=4):** The study design chosen was not appropriate for the research question **OR** Inappropriate statistical analyses were applied to assess the research questions.
   5. **Not rated/applicable:** Do not select for this metric.
2. **Statistical power (sensitivity)**
   1. **High (score=1):** Do not select for this metric.
   2. **Medium (score=2):** The number of participants are adequate to detect an effect in the exposed population and/or subgroups of the total population **OR** The paper reported statistical power is high enough (≥ 80%) to detect an effect in the exposure population and/or subgroups of the total population.
   3. **Low (score=3):** Do not select for this metric.
   4. **Unacceptable (score=4):** The number of participants is inadequate to detect an effect in the exposed population and/or subgroups of the total population
   5. **Not rated/applicable:** Do not select for this metric.
3. **Reproducibility of analyses**
   1. **High (score=1):** Do not select for this metric.
   2. **Medium (score=2):** The description of the analysis is sufficient to understand precisely what has been done and to be conceptually reproducible with access to the analytic data.
   3. **Low (score=3):** The description of the analysis is insufficient to understand what has been done and to be reproducible **OR** a description of analyses are not present (e.g., statistical tests and estimation procedures were not described, variables used in the analysis were not listed, transformations of continuous variables (e.g., logarithmic) were not explained, rules for categorization of continuous variables were not presented, exclusion of outliers was not elucidated and how missing values are dealt with was not mentioned).
   4. **Unacceptable (score=4):** Do not select for this metric.
   5. **Not rated/applicable:** Do not select for this metric.
4. **Statistical Models (confounding bias)**
   1. **High (score=1):** Do not select for this metric.
   2. **Medium (score=2):** The model or method for calculating the risk estimates (e.g., odds ratios, SMRs, SIR) is transparent (i.e., it is stated how/why variables were included or excluded) **AND** Model assumptions were met.
   3. **Low (score=3):** The statistical model building process is not fully appropriate **OR** model assumptions were not met **OR** a description of analyses are not present [STROBE Checklist 12e (Von Elm et al., 2008)].
   4. **Unacceptable (score=4):** Do not select for this metric.
   5. **Not rated/applicable:** Enter ‘NA’ if the study did not use a statistical model.

**Domain 6. Other (if applicable) Considerations for Biomarker Selection and Measurement**

1. **Use of Biomarker of Exposure (detection/measurement/information biases)**
   1. **High (score=1):** Biomarker in a specified matrix has accurate and precise quantitative relationship with external exposure, internal dose, or target dose **AND** Biomarker is derived from exposure to one parent chemical.
   2. **Medium (score=2):** Biomarker in a specified matrix has accurate and precise quantitative relationship with external exposure, internal dose, or target dose **AND** Biomarker is derived from multiple parent chemicals.
   3. **Low (score=3):** Evidence exists for a relationship between biomarker in a specified matrix and external exposure, internal dose or target dose, but there has been no assessment of accuracy and precision or none was reported.
   4. **Unacceptable (score=4):** Biomarker in a specified matrix is a poor surrogate (low accuracy, specificity, and precision) for exposure/dose.
   5. **Not rated/applicable:** Enter ‘NA’ and do not score the metric if no biomarker of exposure was measured.
2. **Effect biomarker (detection/measurement/information biases)**
   1. **High (score=1):** Effect biomarker measured is an indicator of a key event in an adverse outcome pathway (AOP)
   2. **Medium (score=2):** Biomarkers of effect shown to have a relationship to health outcomes using well validated methods, but the mechanism of action is not understood.
   3. **Low (score=3):** Biomarkers of effect shown to have a relationship to health outcomes, but the method is not well validated and mechanism of action is not understood.
   4. **Unacceptable (score=4):** Biomarker has undetermined consequences (e.g., biomarker is not specific to a health outcome).
   5. **Not rated/applicable:** Enter ‘NA’ and do not score the metric if no biomarker of effect was measured.
3. **Method sensitivity (detection/measurement/information biases)**
   1. **High (score=1):** Do not select for this metric.
   2. **Medium (score=2):** Limits of detection are low enough to detect chemicals in a sufficient percentage of the samples to address the research question. Analytical methods measuring biomarker are adequately reported. The limit of detection (LOD) and limit of quantification (LOQ) (value or %) are reported.
   3. **Low (score=3):** Frequency of detection too low to address the research hypothesis **OR** LOD/LOQ (value or %) are not stated
   4. **Unacceptable (score=4):** Do not select for this metric.
   5. **Not rated/applicable:** Enter ‘NA’ and do not score the metric.
4. **Biomarker stability (detection/measurement/information biases)**
   1. **High (score=1):** Samples with a known storage history and documented stability data or those using real-time measurements.
   2. **Medium (score=2):** Samples have known losses during storage, but the difference between low and high exposures can be qualitatively assessed.
   3. **Low (score=3):** Samples with either unknown storage history and/or no stability data for target analytes and high likelihood of instability for the biomarker under consideration
   4. **Unacceptable (score=4):** Do not select for this metric.
   5. **Not rated/applicable:** Enter ‘NA’ and do not score the metric if no biomarkers were assessed.
5. **Sample contamination (detection/measurement/information biases)**
   1. **High (score=1):** Samples are contamination-free from the time of collection to the time of measurement (e.g., by use of certified analyte free collection supplies and reference materials, and appropriate use of blanks both in the field and lab) **AND** Documentation of the steps taken to provide the necessary assurance that the study data are reliable is included.
   2. **Medium (score=2):** Samples are stated to be contamination-free from the time of collection to the time of measurement **AND** There is incomplete documentation of the steps taken to provide the necessary assurance that the study data are reliable.
   3. **Low (score=3):** Samples are known to have contamination issues, but steps have been taken to address and correct contamination issues **OR** Samples are stated to be contamination-free from the time of collection to the time of measurement, but there is no use or documentation of the steps taken to provide the necessary assurance that the study data are reliable.
   4. **Unacceptable (score=4):** There are known contamination issues and no documentation that the issues were addressed.
   5. Enter ‘NA’ and do not score the metric if no samples were collected.
6. **Method requirements (detection/measurement/information biases)**
   1. **High (score=1):** Instrumentation that provides unambiguous identification and quantitation of the biomarker at the required sensitivity [e.g., gas chromatography/high resolution mass spectrometry (GC–HRMS); gas chromatography with tandem mass spectrometry (GC–MS/MS); liquid chromatography with tandem mass spectrometry (LC–MS/MS)].
   2. **Medium (score=2):** Instrumentation that allows for identification of the biomarker with a high degree of confidence and the required sensitivity [e.g., gas chromatography mass spectrometry (GC–MS), gas chromatography with electron capture detector (GC–ECD)].
   3. **Low (score=3):** Instrumentation that only allows for possible quantification of the biomarker, but the method has known interferants [e.g., gas chromatography with flame-ionization detection (GC–FID), spectroscopy].
   4. **Unacceptable (score=4):** Do not select for this metric.
   5. **Not rated/applicable:** Enter ‘NA’ and do not score the metric if no biomarkers were assessed.
7. **Matrix adjustment (detection/measurement/information biases)**
   1. **High (score=1):** If applicable for the biomarker under consideration, study provides results, either in the main publication or as a supplement, for both adjusted and unadjusted matrix concentrations (e.g., creatinine-adjusted or specific gravity-adjusted and non-adjusted urine concentrations) and reasons are given for adjustment approach.
   2. **Medium (score=2):** If applicable for the biomarker under consideration, study only provides results using one method (matrix-adjusted or not).
   3. **Low (score=3):** If applicable for the biomarker under consideration, no established method for matrix adjustment was conducted.
   4. **Unacceptable (score=4):** Do not select for this metric.
   5. **Not rated/applicable:** Enter ‘NA’ and do not score the metric if no biomarkers were assessed.

To determine the overall study score, the first step is to multiply the score for each metric (1, 2, or 3 for high, medium, or low confidence, respectively) by the appropriate weighting factor, as shown in Table S2, to obtain a weighted metric score. The weighted metric scores are then summed and divided by the sum of the weighting factors (for all metrics that are scored) to obtain an overall study score between 1 and 3. The equation for calculating the overall score is shown below:

*Overall Score (range of 1 to 3) = Σ (Metric Score × Weighting Factor)/Σ (Weighting Factors)*

Studies with any single metric scored as unacceptable (score = 4) will be automatically assigned an overall quality score of 4 (unacceptable) and further evaluation of the remaining metrics is not necessary. An unacceptable score means that serious flaws are noted in the domain metric that consequently make the data unusable (or invalid). EPA/OPPT plans to use data with an overall quality level of *High, Medium*, or *Low* confidence to quantitatively or qualitatively support the risk evaluations, but does not plan to use data rated as *Unacceptable*.

Any metrics that are *not rated/not applicable* to the study under evaluation will not be considered in the numerator or calculation of the study’s overall quality score. These metrics will not be included in the nominator or denominator of the *overall score* equation. The overall score will be calculated using only those metrics that receive a numerical score. In addition, if a publication reports more than one study or endpoint, each study and, as needed, each endpoint will be evaluated separately.

Table S2. Metric Weighting Factors and Range of Weighted Metric Scores for Scoring the Quality of Epidemiology Studies.

| **Domain Number/**  **Description** | **Metric Number/Description** | **Range of Metric Scores** | **Metric Weighting**  **Factor** | Domain Weight | **Range of Weighted Metric Scores** |
| --- | --- | --- | --- | --- | --- |
| Study Population | Participant Selection* | 1 to 3 | 0.4 | 1 | 0.4 to 1.2 |
|  | Attrition* | 1 to 3 | 0.4 |  | 0.4 to 1.2 |
|  | Comparison Group | 1 to 3 | 0.2 |  | 0.2 to 0.6 |
| Exposure Characterization | Measurement of Exposure* | 1 to 3 | 0.4 | 1 | 0.4 to 1.2 |
|  | Exposure Levels | 1 to 3 | 0.2 |  | 0.2 to 0.6 |
|  | Temporality* | 1 to 3 | 0.4 |  | 0.4 to 1.2 |
| Outcome Assessment | Outcome measurement or characterization* | 1 to 3 | 0.67 | 1 | 0.67 to 2.01 |
|  | Reporting Bias | 1 to 3 | 0.33 |  | 0.33 to 0.99 |
| Potential Confounding/  Variable Control | Covariate Adjustment* | 1 to 3 | 0.5 | 1 | 0.5 to 1.5 |
|  | Covariate Characterization | 1 to 3 | 0.25 |  | 0.25 to 0.75 |
|  | Co-exposure Confounding/Moderation/  Mediation | 1 to 3 | 0.25 |  | 0.25 to 0.75 |
| Analysis | Study Design and Methods* | 1 to 3 | 0.4 | 1 | 0.4 to 1.2 |
|  | Statistical Power | 1 to 3 | 0.2 |  | 0.2 to 0.6 |
|  | Reproducibility of Analyses | 1 to 3 | 0.2 |  | 0.2 to 0.6 |
|  | Statistical Models | 1 to 3 | 0.2 |  | 0.2 to 0.6 |
| Other  (if applicable) Considerations for Biomarker Selection and Measurement | Use of Biomarker of Exposure | 1 to 3 | 0.143 | 1 | 0.143 to 0.429 |
|  | Effect Biomarker | 1 to 3 | 0.143 |  |  |
|  | Method Sensitivity | 1 to 3 | 0.143 |  |  |
|  | Biomarker Stability | 1 to 3 | 0.14 |  |  |
|  | Sample Contamination | 1 to 3 | 0.143 |  |  |
|  | Method Requirements | 1 to 3 | 0.143 |  |  |
|  | Matrix Adjustment | 1 to 3 | 0.143 |  |  |
| Overall Score = Sum of Weighted Scores/Sum of Metric Weighting Factor | | | | *Sum of Weighted*  *Scores = 6 to 18*  *Sum of Metric Weighting*  *Factors= 6*  6/6=1;  18/6=3  Range of overall  score = 1 to 3  Overall Score = Sum of Weighted Scores/Sum of Metric Weighting Factor | |

*****Indicates “critical” metrics

Table S3. Risk of bias ratings using the Adgent et al. (2014) study

| **OHAT** | | |
| --- | --- | --- |
| **Domain** | **Rating** | **Justification** |
| Selection bias | Probably low risk of bias | Original cohort was convenience sample. Parents may have been incentivized to participate in the postpartum study if their child exhibited cognitive or behavioral issues, however, PBDE levels did not appear to be too different (at least the medians were comparable across groups) |
| Confounding bias | Probably high risk of bias | Confounders included child sex, maternal age, parity, education, maternal race, breastfeeding duration, postpartum income, breast milk omega 3 fatty acid concentration, and fatty acid assay batch, HOME scores, maternal stress. |
| Attrition/Exclusion bias | Probably high risk of bias | Only 64% of eligible women participated, and only 73% of those continued on to the postpartum part of the study. |
| Detection bias-Exposure characterization | Definitely low risk of bias | Breast milk collected 3 months postpartum and PBDE levels assessed at CDC lab. |
| Detection bias- Outcome characterization | Probably low risk of bias | Behavioral development assessed at 36 months using the parent rating scale for preschool-age children of the BASC-2. Cognitive development assessed with the Mullen Scales of Early Learning. Scales were validated for children at this age, but parental report may be subject to bias despite not knowing exposure levels if they are correlated with parental characteristics or if perception of child behavior is associated with exposure sources. |
| Selective reporting bias | Definitely low risk of bias | All outcomes described in methods were reported. Sufficient detail for meta-analysis. |
| Other sources of bias | Definitely low risk of bias | Statistical methods were appropriate. |
| **IRIS** | | |
| **Domain** | **Rating** | **Justification** |
| Exposure measurement | Good | Timing was etiologically relevant, analysis was done at CDC lab. |
| Outcome ascertainment | Good | BASC-II was validated for ages 2 and above |
| Participant selection | Adequate | No information was given about initial recruitment, who may have participated due to concerns re: exposure or neurodevelopment. Demographic differences b/w those lost to follow up and those retained. |
| Confounding | Deficient | Did not have all Tier I confounders |
| Analysis | Good | There is sufficient attention to modeling assumptions and the analysis strategy is well-motivated. |
| Selective reporting | Good | All proposed analyses were presented, reporting of results. Presentation of results not based on statistical significance. |
| Sensitivity | Deficient | The sample size may be too small to detect an effect. Some of the PBDE measures also have minimal variation across subjects. |
| Overall study confidence | Low | One or more deficient ratings |
| **TSCA** | | |
| **Domain** | **Rating** | **Justification** |
| Participant selection | 2 - Medium |  |
| Attrition | 3 - Low | High proportion of missing data. Did not do imputation or any other method to address the missingness. Outcome data could have been associated with loss to follow up, even though exposure was not differential. |
| Comparison group | 1 - High | Key elements of the study design were reported; they were recruited from the same source population. However, demographics are not reported by groups so balance is difficult to ascertain. |
| Measurement of exposure | 1 - High | Exposure was consistently measured using well-established methods. |
| Exposure levels | 2 - Medium | Could not select high for this metric. |
| Temporality | 1 - High | Breast milk PBDE exposure precedes child neurodevelopment at age 3. The interval is a relevant time window for exposure. |
| Outcome measurement or characterization | 1 - High | The outcome measurement was done using well-established methods with a parent questionnaire. Not sure if these are considered the gold standard, but they are validated. So Medium did not seem like a correct rating. |
| Reporting bias | 1 - High | All outcomes were reported, and associations with confidence intervals were presented. |
| Covariate adjustment | 3 - Low | Did not include marital status, alcohol use, depression, tobacco exposure, or exposure to other neurotoxic agents |
| Covariate characterization | 2 - Medium | Breast milk fatty acid procedure was not validated, but the gold standard for child sex and race is self-report. Also, HOME inventory and PSS are validated questionnaires. |
| Co-exposure confounding | 3 - Low | Phthalates could be a co-exposure that was not measured and may bias the findings |
| Study design and methods | 2 - Medium | Could not select high or low for this metric. The cohort study design was appropriate for the research design, and the statistical methods were appropriate. |
| Statistical power | 4 - Unacceptable | Could not select high or low for this metric. Given the observed effect size and the confidence interval widths, it appears the study did not have enough participants to detect a statistically significant effect. |
| Reproducibility of analyses | 2 - Medium | Could not select high or unacceptable for this metric. The analysis is described thoroughly. How variables were categorized is not clearly described, but other than that, all relevant information appears to have been included. |
| Statistical models | 3 - Low | Model assumptions were not described. * We think they were, but they don't explicitly say what the model assumptions are. |
| Use of biomarker of exposure | 1 - High |  |
| Effect biomarker | NA | No biomarker of effect used |
| Method sensitivity | 2 - Medium | Could not select high or unacceptable for this metric. The limits of detection were low enough to detect chemicals in enough samples. The methods are adequately reported. |
| Biomarker stability | 3 - Low | Stability data is not reported and unclear. |
| Sample contamination | 3 - Low | No discussion of phthalate-free containers, no information about contamination provided |
| Method requirements | 1 - High | The cited paper that discusses the exposure assessment methodology says they used gas chromatography/isotope-dilution high resolution mass spectrometry. Two control and two blank samples were added to each batch of 16 unknown samples for quality control. Laboratory quality assurance practices were regularly monitored |
| Matrix adjustment | 2 - Medium | Breast milk was lipid-normalized, which was done to reduce the variability in lipid content between different milk samples and inhomogeneity originating from lipid separation during freezing. |
| Overall score | 4- Unacceptable | Rated unacceptable because at least one domain was unacceptable |

Table S4. Risk of bias ratings using the Chao et al. (2011) study

| **OHAT** | | |
| --- | --- | --- |
| **Domain** | **Rating** | **Justification** |
| Selection bias | Probably low risk of bias | Original cohort was selected with same exclusion and inclusion criteria for exposed and unexposed. A significant portion was excluded due to lack of data, but not clear if this could be differential by exposure or outcome. |
| Confounding bias | Probably high risk of bias | Confounders included maternal age, prepregnancy BMI, gestational age, and infant age at testing. Smoking was controlled via the exclusion criteria. |
| Attrition/Exclusion bias | Probably high risk of bias | Percent excluded due to missing information or formula-fed was almost 50%, but no comparison of maternal characteristics was done to identify whether they differed in important ways. |
| Detection bias-Exposure characterization | Probably low risk of bias | Use of home freezers could've introduced bias |
| Detection bias- Outcome characterization | Probably high risk of bias | There is indirect evidence from other studies that the outcome was assessed using an insensitive instrument (Bayley-III for infants between 8 and 12 months old) |
| Selective reporting bias | Definitely high risk of bias | Only significant associations of breast milk PBDEs with infant neurodevelopment using multiple stepwise linear regression are reported |
| Other sources of bias | Probably low risk of bias | Correlations between PBDEs and neurodevelopment and stepwise linear regression was done. More discussion of the distribution of variables and appropriate model selection could've been done. |
| **IRIS** | | |
| **Domain** | **Rating** | **Justification** |
| Exposure measurement | Good | Seems like a standard approach of measuring exposure, and differential misclassification seems unlikely. |
| Outcome ascertainment | Critically deficient | Unclear if Bayley-III was validated, and if so, in what population. Prior studies indicate it over estimates development and therefore underestimates developmental delay |
| Participant selection | Deficient | Participation was determined by whether exposure was present. Participation could have been influenced by whether parents felt their child may have neurodevelopmental delay. There was no comparison of characteristics of those who were included versus excluded. |
| Confounding | Deficient | Did not have all Tier I confounders |
| Analysis | Deficient | No discussion of missing data, beyond those who did not have exposure or outcome measurements, who were excluded. Results presented without confidence intervals, just significant results presented. No sensitivity analyses. |
| Selective reporting | Deficient | Only significant adjusted results are reported |
| Sensitivity | Deficient | May not have had enough range in the exposure to detect an effect. Sample size may have been too small to detect an effect. |
| Overall study confidence | Uninformative | One or more critically deficient ratings |
| **TSCA** | | |
| **Domain** | **Rating** | **Justification** |
| Participant selection | 2 - Medium | All key elements of the study design are reported. There is no information about those who didn't participate, but no evidence to indicate participation would be based on PBDE levels. |
| Attrition | 3 - Low | Only 70 women ended up being included out of 350 initially invited to participate. No imputation or other analyses for missing data were done to address the attrition. This could potentially also be rated as unacceptable if the attrition was categorized as large rather than moderate. |
| Comparison group | 1 - High | Key elements of the study design were reported; they were recruited from the same source population. However, demographics are not reported by groups so balance is difficult to ascertain. |
| Measurement of exposure | 1 - High | Exposure was consistently measured using well-established methods that directly measure the exposure. |
| Exposure levels | 3 - Low | The range of exposure in the population is limited. |
| Temporality | 1 - High | Breast milk PBDE exposure precedes child neurodevelopment at age 8-12 months. The interval is a relevant time window for exposure. |
| Outcome measurement or characterization | 3 - Low | The Bayley-III has documented limitations for children below 2 years of age. |
| Reporting bias | 3 - Low | Only statistically significant results are presented for the adjusted analyses. |
| Covariate adjustment | 3 - Low | Did not include HOME inventory, maternal education, marital status, alcohol use, depression, poverty, child sex, or exposure to other neurotoxic agents |
| Covariate characterization | 2 - Medium | Confounders included maternal age, prepregnancy BMI, gestational age, and infant age at time of testing. These were collected via questionnaire. |
| Co-exposure confounding | 3 - Low | Could not select high or unacceptable for this metric. No mention of co-exposures, and it is possible that phthalates could have co-occurred. |
| Study design and methods | 2 - Medium | Could not select high for this metric. The study design was appropriate and statistical methods were appropriate. |
| Statistical power | 4 - Unacceptable | Could not select high or low for this metric. Given the observed effect size and the reported p-values, it appears the study did not have enough participants to detect a statistically significant effect at the observed effect levels. |
| Reproducibility of analyses | 2 - Medium | Could not select high or unacceptable for this metric. The description of analysis is sufficient, and is conceptually reproducible. |
| Statistical models | 3 - Low | Could not select high or unacceptable for this metric. The model assumptions were not described, so cannot determine if they were met. |
| Use of biomarker of exposure | 1 - High | Biomarker of PBDE exposure in breast milk likely has precise quantitative relationship with exposure to PBDEs, but I'm not sure how to verify and/or learn that. I assume it is derived from exposure to one parent chemical, but don't really know what that m |
| Effect biomarker | NA |  |
| Method sensitivity | 2 - Medium | If the method is sensitive enough so that a high proportion of subjects exceed the LOD, this is satisfied. The actual value of the LOD does not need to be reported. |
| Biomarker stability | 3 - Low | Could not select high or unacceptable for this metric. The limits of detection were low enough to detect chemicals in enough samples. The methods are adequately reported. |
| Sample contamination | 2 - Medium | Could not select unacceptable for this metric. Sample had known storage history, but no stability information. |
| Method requirements | 1 - High | Study states the breast milk samples were collected in chemical-free glass bottles, but no further information was provided about steps taken to ensure the study data were reliable. |
| Matrix adjustment | 2 - Medium | Study used high-resolution gas chromatograph and high-resolution mass spectrometer. |
| Overall score | 4- Unacceptable | Rated unacceptable because at least one domain was unacceptable |

Table S5. Risk of bias ratings using the Chen et al. (2014) study

| **OHAT** | | |
| --- | --- | --- |
| **Domain** | **Rating** | **Justification** |
| Selection bias | Probably low risk of bias | Participants were recruited from the same population and had the same exclusion criteria. It is not known whether they had the same participation rates. |
| Confounding bias | Probably high risk of bias | Did not include alcohol |
| Attrition/Exclusion bias | Probably low risk of bias | There were differences between the 309 women included and the 80 excluded, but this was about 20%. Furthermore, the child participants who had an IQ test at age 5 were not statistically different in any characteristics from those that did not have an IQ test at age 5. |
| Detection bias-Exposure characterization | Definitely low risk of bias | The exposure was measured by a CDC lab using well-established methods |
| Detection bias- Outcome characterization | Probably low risk of bias | The outcome was measured using validated instruments, but not a gold standard. |
| Selective reporting bias | Definitely low risk of bias | All measured outcomes reported |
| Other sources of bias | Definitely low risk of bias | Non-linear associations and interactions were assessed, sensitivity analyses showed similar results. |
| **IRIS** | | |
| **Domain** | **Rating** | **Justification** |
| Exposure measurement | Good | Serum samples from early pregnancy were analyzed for 10 PBDE congener levels. Analysis was done at the CDC's Persistent Organic Pollutants Biomonitoring Laboratory at the National Center for Environmental Health. |
| Outcome ascertainment | Adequate | Bayley-II, BASC-2, and Weschler for IQ which are validated for ages. Marked down from good because the BASC-2 is not validated for age 1. |
| Participant selection | Deficient | 80 women were excluded from the original cohort due to missing exposure or outcomes. Difference in characteristics was noted in text but data was not shown. Those missing IQ test at 5 years were not different from those in demographics from those who were. Cohort was a convenience sample, don't know how many women were offered the opportunity to be included in the study but refused. However, given that they were recruited in early pregnancy, they couldn't have volunteered based on knowledge of outcome (and unlikely for exposure, too). |
| Confounding | Adequate | Had all Tier I confounders but not Tier II |
| Analysis | Good | The following confounders were not included: maternal use of alcohol during pregnancy, gestational exposure to environmental tobacco smoke, birth weight or gestational age, number of children in the home, father's presence in the home, preschool and out-of-home child care facility attendance, psychometrician, location and language of the assessment. The following confounders were included that are not on the list: maternal race/ethnicity, maternal IQ. |
| Selective reporting | Good | Transformations and non-linearities were considered and implemented when necessary. Appropriate consideration of longitudinal design. Covariates identified a priori. Descriptive information about exposure and outcome presented. Quantitative results with confidence intervals are presented. Missing data noted and compared to full sample. Sensitivity analyses completed. |
| Sensitivity | Adequate | No indication of selective reporting; all analyses mentioned are presented |
| Overall study confidence | Low | One or more deficient ratings |
| **TSCA** | | |
| **Domain** | **Rating** | **Justification** |
| Participant selection | 1 - High | Key elements of the study design were reported. Possible lead paint exposure was an inclusion criterion, which may make the cohort less generalizable but allows for assessment of other potential neurotoxins. |
| Attrition | 3 - Low | 190 out of a total of 309 collected infant information. The outcomes were not imputed, although the participants were not different from those lost to follow up in demographics or PBDE levels |
| Comparison group | 1 - High | Decided not to mark down if they didn't create categories for PBDE exposure. Otherwise, subjects were recruited from the same population at the same time with the same inclusion and exclusion criteria. |
| Measurement of exposure | 1 - High | Exposure was consistently measured using well-established methods that directly measure the exposure. |
| Exposure levels | 2 - Medium | Could not select high for this metric. There is sufficient variation in the exposure and was observed at a relatively high level. |
| Temporality | 1 - High | Maternal serum PBDE levels preceded child neurodevelopment outcomes, and prenatal exposure is considered a relevant exposure window |
| Outcome measurement or characterization | 1 - High | Outcome measure was validated in US cohort. |
| Reporting bias | 1 - High | All outcomes were reported, and associations with confidence intervals were presented. |
| Covariate adjustment | 3 - Low | Did not include alcohol |
| Covariate characterization | 3 - Low | No mention of how covariates were collected or measured. |
| Co-exposure confounding | 2 - Medium | Measured maternal blood lead levels and examined as potential co-exposure. |
| Study design and methods | 2 - Medium | Could not select high or low for this metric. Study design was appropriate and statistical methods were sound. |
| Statistical power | 4 - Unacceptable | Could not select high or low for this metric. The number of people seems insufficient for some outcomes, but not all. Not sure how to rate. |
| Reproducibility of analyses | 2 - Medium | Could not select high or unacceptable for this metric. The description of analysis is sufficient, and is conceptually reproducible. |
| Statistical models | 3 - Low | Decided to mark as low if model assumptions were not described. |
| Use of biomarker of exposure | 1 - High | Biomarker of PBDE exposure in maternal serum during pregnancy |
| Effect biomarker | NA |  |
| Method sensitivity | 2 - Medium | Could not select high or unacceptable for this metric. The limits of detection were low enough to detect chemicals in enough samples. The methods are adequately reported. |
| Biomarker stability | 3 - Low | Could not select unacceptable for this metric. Sample had known storage history, but no stability information. |
| Sample contamination | 3 - Low | Decided to rate as low if sample containers were not described at all. |
| Method requirements | 1 - High | Gas chromatography/isotope dilution high-resolution mass spectrometry was done. |
| Matrix adjustment | 2 - Medium | Samples were lipid-adjusted, non-lipid adjusted results were not shown. |
| Overall score | 4- Unacceptable | Rated unacceptable because at least one domain was unacceptable |

Table S6. Risk of bias ratings using the Cowell et al. (2015) study

| **OHAT** | | |
| --- | --- | --- |
| **Domain** | **Rating** | **Justification** |
| Selection bias | Probably low risk of bias | Women were recruited from New York City hospitals if they had been pregnant during the 9/11 attacks, and were still pregnant between December 2001 and January 2002. Recruitment did not differ based on exposure status, although it is possible that the most strongly exposed were subject to selection if exposure affected risk of miscarriage or stillbirth. However, the inclusion and exclusion criteria were the same. |
| Confounding bias | Probably high risk of bias | Covariates did not include HOME inventory, maternal education, alcohol use, household income, and exposure to other neurotoxic agents |
| Attrition/Exclusion bias | Probably high risk of bias | Only about 2/3 of women originally enrolled had cord blood available, and while the authors present differences in the whole cohort versus those with cord blood, they don't compare to those missing. This suggests there may be stronger differences between groups than is reported. |
| Detection bias-Exposure characterization | Definitely low risk of bias | The exposure was consistently assessed using a CDC lab. |
| Detection bias- Outcome characterization | Probably low risk of bias | Outcome was assessed using validated methods, but they aren't the gold standard. |
| Selective reporting bias | Definitely low risk of bias | All measured outcomes reported |
| Other sources of bias | Definitely low risk of bias | Appropriate statistical models were used, non-linear associations were assessed, effect modification was examined. |
| **IRIS** | | |
| **Domain** | **Rating** | **Justification** |
| Exposure measurement | Good | Analyzed at CDC's lab. |
| Outcome ascertainment | Good | The Child Behavior Checklist was administered for the child at age 3 years through age 7 years. It is validated for use among preschool and school-aged children. It has a mother rate her child's behavior. |
| Participant selection | Deficient | Women recruited from hospitals in New York. Women had to be between 18-39, smoked less than one cigarette per day, did not use illegal drugs, and not have preexisting medical condition. Unclear how many people were approached. Most exposed may have moved out of New York area after 9/11 or potentially miscarried. Loss to follow up was reported and characteristics were compared among those retained and those lost. |
| Confounding | Deficient | Did not have all Tier I confounders |
| Analysis | Adequate | Did not expect effect of PBDEs on child attention to vary over time, so didn't analyze data using repeated measures. However, other studies suggest the effect does change over time. Used negative binomial model because Poisson was over-dispersed. Did not look for non-linearity. Missing data are acknowledged and accounted for in analysis. Analysis stratifies by infant sex because of a priori hypothesis that there could be sex-specific effects. |
| Selective reporting | Good | No indication of selective reporting; all analyses mentioned are presented |
| Sensitivity | Adequate | Good variability in exposure and outcome. Small sample size |
| Overall study confidence | Low | One or more deficient ratings |
| **TSCA** | | |
| **Domain** | **Rating** | **Justification** |
| Participant selection | 2 - Medium | Key elements of study design are reported, and the inclusion criteria. However, people may have miscarried as a result of being exposed to the WTC attacks, and therefore it is possible that those women whose pregnancies persisted are not representative of those who would've been eligible. |
| Attrition | 4 - Unacceptable | Large subject attrition. |
| Comparison group | 1 - High | Decided not to mark down if they didn't create categories for PBDE exposure. Otherwise, subjects were recruited from the same population at the same time with the same inclusion and exclusion criteria. |
| Measurement of exposure | 1 - High | Exposure was consistently measured using well-established methods that directly measure the exposure. |
| Exposure levels | 3 - Low | The Median and IQR are presented, but the IQR is just presented as 1 number so hard to interpret. The min and max are not shown. |
| Temporality | 1 - High | Cord blood PBDEs come before child neurodevelopment at age 4 and 6, and is a relevant exposure window. |
| Outcome measurement or characterization | 2 - Medium | The outcome measurement was done using well-established methods, although not a gold standard. |
| Reporting bias | 3 - Low | Did not report the subscales, and did not report the results from the earlier ages. |
| Covariate adjustment | 3 - Low | Did not include HOME inventory, education, alcohol use, poverty |
| Covariate characterization | 2 - Medium | Reproductive history should've been collected from the medical record rather than self-report. |
| Co-exposure confounding | 3 - Low | Could not select high or unacceptable for this metric. There was no discussion of other exposures. |
| Study design and methods | 4 - Unacceptable | Could not select high or low for this metric. The statistical methods were not appropriate to assess the research question, because they had repeated measures but did not analyze their data with this in mind. However, they do use robust standard errors which should address clustering. However, there are potential time-varying confounders that are not adequately modeled. |
| Statistical power | 4 - Unacceptable | Could not select high or low for this metric. Given the observed effect size and the confidence interval widths, it appears the study did not have enough participants to detect a statistically significant effect. |
| Reproducibility of analyses | 2 - Medium | Could not select high or unacceptable for this metric. The description of analysis is sufficient, and is conceptually reproducible. |
| Statistical models | 3 - Low | Decided to mark as low if model assumptions were not described. |
| Use of biomarker of exposure | 1 - High | Biomarker of PBDE exposure in cord blood at delivery had quantitative relationship with exposure |
| Effect biomarker | NA |  |
| Method sensitivity | 3 - Low | Only 50% were above the LOD for PBDE-153 |
| Biomarker stability | 3 - Low | Could not select unacceptable for this metric. Sample had known storage history, but no stability information. |
| Sample contamination | 2 - Medium | Cited paper says that the containers were contamination-free. |
| Method requirements | 1 - High | Gas chromatography/isotope dilution high-resolution mass spectrometry was done. |
| Matrix adjustment | 2 - Medium | Samples were lipid-adjusted, non-lipid adjusted results were not shown. |
| Overall score | 4- Unacceptable | Rated unacceptable because at least one domain was unacceptable |

Table S7. Risk of bias ratings using the Eskenazi et al. (2013) study

| **OHAT** | | |
| --- | --- | --- |
| **Domain** | **Rating** | **Justification** |
| Selection bias | Probably low risk of bias | Recruitment was the same for exposed and non-exposed, with the same exclusion criteria, and from the same time period. |
| Confounding bias | Definitely low risk of bias | All tier 1 and 2 confounders included |
| Attrition/Exclusion bias | Probably high risk of bias | 63 children lacked PBDE measurements, but their maternal PBDE prenatal PBDE measurements did not significantly differ from those who were included. |
| Detection bias-Exposure characterization | Definitely low risk of bias | The exposure was consistently addressed using well-established methods from CDC lab |
| Detection bias- Outcome characterization | Probably low risk of bias | The outcomes were measured using validated instruments, but not a gold standard. |
| Selective reporting bias | Definitely low risk of bias | All measured outcomes reported |
| Other sources of bias | Definitely low risk of bias | Statistical methods were appropriate, non-linearities were assessed, effect modification was examined. |
| **IRIS** | | |
| **Domain** | **Rating** | **Justification** |
| Exposure measurement | Good | PBDE exposure was assessed using maternal serum either during pregnancy or at delivery. Children's samples were collected at the 7-year visit. Samples were analyzed at the CDC lab. Strategy for dealing with values below LOD seems reasonable. |
| Outcome ascertainment | Adequate | Neurobehavioral assessments were performed by bilingual psychometricians. Attention was measured at the 5-year visit by the Child Behavior Checklist which was completed by mothers. At 7 years, mothers and teachers completed the Conners' ADHD/DMS-Iv scales and the Behavior Assessment System for Children-2nd edition. Motor function was assessed at ages 5 and 7 years using the McCarthy Scales of Children's Abilities. Cognitive functioning was assessed at 5 years using the PPVT and TVIP, and performance intelligence was assessed using the Wechsler Preschool and Primary Scale of Intelligence, 3rd edition. At 7 years, children were assessed using Wechsler Intelligence Scale for Children-4th edition. A full scale IQ was also calculated. |
| Participant selection | Deficient | To be eligible, women had to be at least 18 years old, less than 20 weeks gestation, Spanish or English speaking, qualifying for low-income health insurance, and planning to deliver at the public hospital. 601 women were included, 526 delivered live born singletons. Excludes 4 children with autism, down syndrome, cerebral palsy/hydrocephaulus, or deafness. Also excludes 63 children who did not have PBDE measurements. Compared with children in the cohort not followed, those who were included tended to have a few demographic differences but did not differ by maternal prenatal PBDE levels. |
| Confounding | Good | Confounders included maternal age, education, years in US, marital status, work outside the home, use of tobacco and alcohol during pregnancy, depression, parity, PPVT or TVIP score, housing density, household poverty, pregnancy exposure to environmental tobacco smoke, number of children in the home, father's presence, HOME score, psychometrician, location and language of the assessment, child sex, birth weight, ptb status, handedness |
| Analysis | Adequate | No descriptive tables or information |
| Selective reporting | Good | No indication of selective reporting; all analyses mentioned are presented |
| Sensitivity | Deficient | Unknown if there was sufficient range in exposures or outcomes. Sample size could've been bigger |
| Overall study confidence | Low | One or more deficient ratings |
| **TSCA** | | |
| **Domain** | **Rating** | **Justification** |
| Participant selection | 2 - Medium | Pregnant women were eligible if they were at least 18 years old, less than 20 weeks pregnant, Spanish or English speaking, qualifying for low income health insurance, and planning to deliver at the public hospital |
| Attrition | 3 - Low | 323 out of 526 were followed until the children were 7 years old. Nothing was done to address participant attrition besides excluding the lost participants. |
| Comparison group | 1 - High | Decided not to mark down if they didn't create categories for PBDE exposure. Otherwise, subjects were recruited from the same population at the same time with the same inclusion and exclusion criteria. |
| Measurement of exposure | 1 - High | Exposure was consistently measured using well-established methods that directly measure the exposure. |
| Exposure levels | 2 - Medium | Could not select high for this metric. 4 of the 10 PBDE congeners examined had sufficient range to be further examined in the study, and the range and distribution of those were sufficient so that we would expect to observe a relationship should one exist |
| Temporality | 2 - Medium | PBDE measurements were taken at the same time as the neurodevelopment outcomes for a subset of the participants. |
| Outcome measurement or characterization | 2 - Medium | Outcome was measured using well-established methods, but they aren't the gold standard. |
| Reporting bias | 1 - High | Measured outcomes are reported in the methods, and effect estimates are reported with a confidence interval. |
| Covariate adjustment | 1 - High | All tier 1 and tier 2 confounders included. |
| Covariate characterization | 2 - Medium | Alcohol and smoking were assessed on self-report (we presume), depression was assessed using validated questionnaire. |
| Co-exposure confounding | 2 - Medium | Co-exposures were controlled for in sensitivity analyses. |
| Study design and methods | 2 - Medium | Could not select high or low for this metric. The study design was appropriate and statistical methods were appropriate. |
| Statistical power | 4 - Unacceptable | Could not select high or low for this metric. Sample size was large enough to detect effects for some exposure-outcome combinations, but not all. |
| Reproducibility of analyses | 2 - Medium | Co-exposures were controlled for in sensitivity analyses. |
| Statistical models | 3 - Low | Decided to mark as low if model assumptions were not described. |
| Use of biomarker of exposure | 1 - High | Could not select high or unacceptable for this metric. The model assumptions were not described, so cannot determine if they were met. |
| Effect biomarker | NA | Biomarker of PBDE exposure in maternal serum during pregnancy, at delivery, or in child's serum at age 7 |
| Method sensitivity | 3 - Low | Detection frequency was greater than 90% for only 4 PBDEs, the others had less than 50% detection frequency. |
| Biomarker stability | 3 - Low | No information is given about sample stability. |
| Sample contamination | 3 - Low | Decided to rate as low if sample containers were not described at all. |
| Method requirements | 1 - High | Gas chromatography/isotope dilution high-resolution mass spectrometry was done. |
| Matrix adjustment | 2 - Medium | Study only provides lipid-adjusted results. |
| Overall score | 4- Unacceptable | Rated unacceptable because at least one domain was unacceptable |

Table S8. Risk of bias ratings using the Gascon et al. (2012) study

| **OHAT** | | |
| --- | --- | --- |
| **Domain** | **Rating** | **Justification** |
| Selection bias | Probably low risk of bias | Recruitment and eligibility were not dependent on exposure status, and were recruited at the same time. |
| Confounding bias | Probably high risk of bias | Did not include HOME inventory, marital status, use of alcohol during pregnancy, maternal depression, or exposure to tobacco smoke. |
| Attrition/Exclusion bias | Definitely high risk of bias | 290 out of 1,295 originally recruited were included in the study, and no comparison of characteristics was done. |
| Detection bias-Exposure characterization | Definitely low risk of bias | Exposure was measured using cited methods, samples were collected from an experienced nurse. Samples were not analyzed at a government lab, however. |
| Detection bias- Outcome characterization | Probably low risk of bias | The outcome was measured using validated instruments, but not a gold standard. Psychologists were specially trained and were not aware of exposure information. |
| Selective reporting bias | Definitely low risk of bias | All measured outcomes reported |
| Other sources of bias | Definitely low risk of bias | Multiple imputation was done to account for missing covariates, variables with non-normal distributions were log-transformed, and the linearity of the associations were assessed. |
| **IRIS** | | |
| **Domain** | **Rating** | **Justification** |
| Exposure measurement | Adequate | Not analyzed in a government lab and QA/QC wasn't compared to anything else |
| Outcome ascertainment | Adequate | Outcome assessment validated but not in Spanish children, which was this study's population |
| Participant selection | Deficient | Proportion of participants approached who agreed to participate is not described. Some were lost to follow-up for reasons that could've been related to exposure or correlated with upstream factors related to exposure levels. Only 290 out of 1,295 total participants were included in this study, and there is no comparison of their characteristics presented. |
| Confounding | Deficient | Confounders included maternal age, social class, education, country of origin, parity, child care attendance, duration of breastfeeding, maternal consumption of fish during pregnancy, maternal pre-pregnancy BMI, child's gestational age, child's weight at birth. For adequate, also need HOME inventory, marital status, maternal depression, child sex. Also, confounders were only included if they were associated with the outcome with a p-value of less than 0.2 or if they changed the coefficient by more than 10%. |
| Analysis | Good | Non-linearity was considered. The outcome variable was transformed when necessary. Descriptive information about the exposure and outcome was presented. The amount of missing data was noted and multiple imputation was performed. |
| Selective reporting | Good | All results described were presented. |
| Sensitivity | Deficient | Range of exposure levels seems sufficient to detect an effect. The population was evaluated at a time period that would be relevant to detection of an effect. The study may not have been adequately powered to detect an effect. |
| Overall study confidence | Low | One or more deficient ratings |
| **TSCA** | | |
| **Domain** | **Rating** | **Justification** |
| Participant selection | 1 - High | All key elements of the study design are reported. Pregnant women were recruited during the first trimester routine antenatal care visit in the main public hospital or health center if they were at least 16 years old, intended to deliver in the city, did not have assisted conception, and were able to communicate with recruiters. |
| Attrition | 4 - Unacceptable | Only 295 out of 1,295 participated. |
| Comparison group | 1 - High | Decided not to mark down if they didn't create categories for PBDE exposure. Otherwise, subjects were recruited from the same population at the same time with the same inclusion and exclusion criteria. |
| Measurement of exposure | 1 - High | Exposure was consistently measured using well-established methods that directly measure the exposure. |
| Exposure levels | 3 - Low | The range of exposure in the population is limited. |
| Temporality | 1 - High | The study presents appropriate temporality and the interval between the exposure and outcome is considerate of the relevant exposure windows. |
| Outcome measurement or characterization | 2 - Medium | The outcome measurement was done using a well-established method (the BSID), although it is not a gold standard. |
| Reporting bias | 1 - High | Effect estimates are presented with confidence intervals. The measured outcomes are outlined in the methods and results are presented in tables. |
| Covariate adjustment | 3 - Low | Did not include HOME inventory, maternal age, marital status, alcohol use, maternal depression. |
| Covariate characterization | 2 - Medium | Smoking is self-report, and the proportion of maternal BMI that is self-report was not stated. |
| Co-exposure confounding | 2 - Medium | Study adjusted for PCBs, DDE, and HCB, and tested for interactions between PBDEs and POPs |
| Study design and methods | 2 - Medium | Study design was appropriate, as were statistical methods. |
| Statistical power | 4 - Unacceptable | Given the observed association size, the sample size was not sufficient to detect an effect. |
| Reproducibility of analyses | 2 - Medium | The description of the analysis is sufficient to be conceptually reproducible. |
| Statistical models | 3 - Low | Decided to mark as low if model assumptions were not described. |
| Use of biomarker of exposure | 1 - High | Biomarker of PBDE exposure in colostrum has quantitative relationship with exposure. |
| Effect biomarker | NA |  |
| Method sensitivity | 3 - Low | Low proportion of subjects had exposure that exceeded the LOD. |
| Biomarker stability | 3 - Low | No information is given about sample stability. |
| Sample contamination | 2 - Medium | In the supplemental material, it is stated that the samples were stored in sterile polypropylene tubes. |
| Method requirements | 2 - Medium | Gas chromatography/mass spectrometry was done, and it was reported to compare well to other methods in international inter-calibration exercises. It is described as liquid-liquid extraction protocol. |
| Matrix adjustment | 2 - Medium | Only lipid-adjusted results are presented. |
| Overall score | 4- Unacceptable | Rated unacceptable because at least one domain was unacceptable |

Table S9. Risk of bias ratings using the Gascon et al. (2011) study

| **OHAT** | | |
| --- | --- | --- |
| **Domain** | **Rating** | **Justification** |
| Selection bias | Probably low risk of bias | Women were recruited when they presented for antenatal care. 94% of eligible mothers were ultimately enrolled. Enrollment and recruitment was not based on exposure. |
| Confounding bias | Probably high risk of bias | Did not include HOME inventory, maternal depression. |
| Attrition/Exclusion bias | Probably high risk of bias | 422 children of 482 in original cohort completed tests at age 4. However, only 88 cord blood samples and 244 serum samples of 4 year old children were done. While the PBDE measurements at age 4 cohort was similar in characteristics to the full cohort, the mothers of children with cord blood measurements had higher social class and education, lower smoking, and differential child behavior outcomes. |
| Detection bias-Exposure characterization | Definitely low risk of bias | Exposure was measured consistently using well-established methods that directly measure exposure |
| Detection bias- Outcome characterization | Probably low risk of bias | Outcome was assessed using validated methods, but they aren't the gold standard. |
| Selective reporting bias | Probably low risk of bias | Did not show results when adjusting for other chemicals |
| Other sources of bias | Probably low risk of bias | Missing covariates were imputed using multiple imputation, and non-linear associations were assessed. Study was underpowered. |
| **IRIS** | | |
| **Domain** | **Rating** | **Justification** |
| Exposure measurement | Adequate | Exposure measurement done at the same time as outcome measurement (4 years of age), may not be etiologically relevant time window |
| Outcome ascertainment | Adequate | Unclear if MSCA and DSM-IV for ADHD are validated for Spanish children |
| Participant selection | Adequate | Women were recruited during antenatal care; 94% of those invited agreed to participate. 98% of those enrolled completed the study through age 4 for their children. Only 88% of the cohort completed the cognitive tests at age 4. |
| Confounding | Deficient | Confounders included sex and age of the child, evaluating psychologist, maternal age, social class, education of the mother, smoking during pregnancy, alcohol consumption, fish consumption, parity, type and duration of lactation, prepregnancy BMI, gestational age at birth and weight at birth. Confounders not included that were necessary for adequate rating included HOME inventory, marital status, maternal depression. |
| Analysis | Adequate | No sensitivity analyses were presented |
| Selective reporting | Adequate | Unclear if they controlled for OC's -- says in the text but not reported in the tables; was a secondary aim |
| Sensitivity | Deficient | Sufficient exposure variability at levels that seem relevant for potential effects to be observed. However, a substantial proportion did not have levels above the LOD. Sample sizes for the exposed group therefore are likely too small for this study to be able to detect an effect of the observed size. |
| Overall study confidence | Low | One or more deficient ratings |
| **TSCA** | | |
| **Domain** | **Rating** | **Justification** |
| Participant selection | 1 - High | All key elements of the study design are reported. Pregnant women presenting for antenatal care were recruited, and 482 (94% of those eligible) were enrolled. |
| Attrition | 3 - Low | Incomplete exposure data because only 88 had cord blood samples out of 482. |
| Comparison group | 1 - High | Differences in baseline characteristics were considered as potential confounders. |
| Measurement of exposure | 1 - High | Exposure was consistently measured using well-established methods that directly measure the exposure. |
| Exposure levels | 3 - Low | The range of exposure in the population is limited. |
| Temporality | 2 - Medium | PBDE in child serum was assessed at 4 years, the same time as the neurodevelopment outcome. |
| Outcome measurement or characterization | 2 - Medium | Outcome was measured using well-established methods, but they aren't the gold standard. |
| Reporting bias | 1 - High | Measured outcomes are reported in the methods, and effect estimates are reported with a confidence interval. Individual outcome components of a composite score are reported. |
| Covariate adjustment | 3 - Low | Did not include HOME inventory, marital status, or depression |
| Covariate characterization | 1 - High | BMI and birthweight and gestational age came from the medical record, everything else came from the questionnaire. |
| Co-exposure confounding | 2 - Medium | Coexposures to pollutants were measured and adjusted for. |
| Study design and methods | 2 - Medium | Study design was appropriate, as were statistical methods. |
| Statistical power | 4 - Unacceptable | Could not select high or low for this metric. Given the observed effect size and the confidence interval widths, it appears the study did not have enough participants to detect a statistically significant effect. |
| Reproducibility of analyses | 2 - Medium | Could not select high for this metric. The description of analysis is sufficient to understand what was done and is conceptually reproducible. |
| Statistical models | 3 - Low | Decided to mark as low if model assumptions were not described. |
| Use of biomarker of exposure | 1 - High | Biomarker of PBDE exposure in cord blood at delivery had quantitative relationship with exposure, as did serum age age 4. |
| Effect biomarker | NA |  |
| Method sensitivity | 3 - Low | Either the exposure levels were too low or the methods were not sensitive enough to be able to detect chemicals in a sufficient portion of the samples. |
| Biomarker stability | 3 - Low | Biomarker of PBDE exposure in cord blood at delivery had quantitative relationship with exposure, as did serum age 4. |
| Sample contamination | 3 - Low | Decided to rate as low if sample containers were not described at all. |
| Method requirements | 2 - Medium | Gas chromatography/ with electron capture detection and chemical ionization negative ion mass spectrometry was done. |
| Matrix adjustment | 3 - Low | Did not report the lipid adjustment methodology, but the tables have the units reported as lipid-adjusted. |
| Overall score | 4- Unacceptable | Rated unacceptable because at least one domain was unacceptable |

Table S10. Risk of bias ratings using the Gump et al. (2014) study

| **OHAT** | | |
| --- | --- | --- |
| **Domain** | **Rating** | **Justification** |
| Selection bias | Probably high risk of bias | While the study does use a simple random sample, the response rate was only 11.2%, and compared to ACS estimates of the study area, the study population was more likely to include more minority participants with slightly lower income. |
| Confounding bias | Probably high risk of bias | Did not include HOME inventory, maternal education, marital status, alcohol use, depression, tobacco smoke. |
| Attrition/Exclusion bias | Probably high risk of bias | While only SES differed between included and excluded participants, more than 50% were excluded, for a sample size of only 43 |
| Detection bias-Exposure characterization | Definitely low risk of bias | QA/QC provided |
| Detection bias- Outcome characterization | Probably high risk of bias | The experimental tasks do not have any information about validation. The psychological tasks were measured using validated instruments that are not the gold standard. |
| Selective reporting bias | Probably high risk of bias | The experimental task results were not reported, it just said the p-values were greater than 0.05. Psychological results are not reported with confidence intervals, only p-values. |
| Other sources of bias | Probably low risk of bias | Linearity of blood PBDE levels was assessed. Sensitivity analyses weren't done, and no effect modification was explored. |
| **IRIS** | | |
| **Domain** | **Rating** | **Justification** |
| Exposure measurement | Good | QA/QC was done and compared to a validated standard |
| Outcome ascertainment | Deficient | Mirror tracing, Go/No Go task, and continuous performance tasks were performed by the children, but these tests, as far as I can tell, are not validated measurements of psychological functioning. The Strengths and Difficulties Questionnaire was also administered (parents answered the questions, which could introduce bias). The authors administered the Buss and Perry aggression scale, the Cook-Medley Ho Scale as a measure of hostility, and the Child Depression Inventory as a measure of depressive symptoms. All outcomes were assessed at the same time as the blood draw for exposure, which may not have been the etiologically relevant time. |
| Participant selection | Critically deficient | Selection bias likely to impact results |
| Confounding | Deficient | Confounders were chosen a prior and included age and sex-standardized BMI percentile, socioeconomic status, total blood lipid levels, lead levels. |
| Analysis | Deficient | PBDE levels were assessed for normality and log-transformed. Linear regression models were used. Results were presented as significant or not significant without confidence intervals. |
| Selective reporting | Good | No evidence of selective reporting, all analyses described in methods were presented. |
| Sensitivity | Deficient | The sample size may be too small to detect an effect. PBDE levels may also have been too low to detect an effect. |
| Overall study confidence | Uninformative | One or more critically deficient ratings |
| **TSCA** | | |
| **Domain** | **Rating** | **Justification** |
| Participant selection | 3 - Low | Participant selection is described, but there was low participation. |
| Attrition | 3 - Low | Exposure data was not complete, only 43 out of 100 had PBDE levels because they changed the hypothesis half-way through. |
| Comparison group | 1 - High | Key elements of the study design were reported and indicate that subjects were recruited from the same eligible population with the same method of ascertainment during the same time period |
| Measurement of exposure | 2 - Medium | QA/QC was modified from another study but the exact modifications were not described. |
| Exposure levels | 3 - Low | Range was too low to be biologically meaningful for some of the congeners. |
| Temporality | 3 - Low | Since PBDEs are persistent it is likely to have occurred prior to the outcome, although it is a cross-sectional study. |
| Outcome measurement or characterization | 2 - Medium | The experimental tasks do not appear to be validated, but the psychological measures are from well-established, validated scales, although they are not the gold standard. |
| Reporting bias | 2 - Medium | All the study's measured outcomes are reported, but confidence intervals are not presented. |
| Covariate adjustment | 3 - Low | Did not include HOME inventory, marital status, alcohol use, depression, or smoking |
| Covariate characterization | 1 - High | Covariates were assessed via questionnaires, and height and weight were measured by study personnel. |
| Co-exposure confounding | 2 - Medium | Lead, a potential neurotoxin, was adjusted for. Other potential co-exposures were not. |
| Study design and methods | 4 - Unacceptable | Cross-sectional study design is not appropriate for the research question. |
| Statistical power | 4 - Unacceptable | Given the observed association size, the sample size was not sufficient to detect an effect for some of the measures, although not all. The sample size was very small, only 43 children. |
| Reproducibility of analyses | 3 - Low | They report standardized betas but do not discuss the methods. There were results whose methods were not described. |
| Statistical models | 3 - Low | Decided to mark as low if model assumptions were not described. |
| Use of biomarker of exposure | 1 - High | Serum PBDE levels have quantitative relationship with exposure. |
| Effect biomarker | NA |  |
| Method sensitivity | 2 - Medium | Detection frequency was greater than 75% for all congeners. |
| Biomarker stability | 3 - Low | Limits of detection are low enough to detect chemicals in a sufficient portion of samples to address the research question. |
| Sample contamination | 2 - Medium | No information is given about sample stability or storage. |
| Method requirements | 1 - High | It is noted that the tubes used for blood storage were pre-certified by the analyzing laboratory. |
| Matrix adjustment | 1 - High | Gas chromatography/ high-resolution mass spectrometry was done. |
| Overall score | 4- Unacceptable | Rated unacceptable because at least one domain was unacceptable |

Table S11. Risk of bias ratings using the Herbstman et al. (2010) study

| **OHAT** | | |
| --- | --- | --- |
| **Domain** | **Rating** | **Justification** |
| Selection bias | Probably low risk of bias | Women were recruited from New York City hospitals if they had been pregnant during the 9/11 attacks, and were still pregnant between December 2001 and January 2002. Recruitment did not differ based on exposure status, although it is possible that the most strongly exposed were subject to selection if exposure affected risk of miscarriage or stillbirth. However, the inclusion and exclusion criteria were the same. |
| Confounding bias | Probably high risk of bias | Did not include HOME inventory, alcohol use, depression |
| Attrition/Exclusion bias | Probably high risk of bias | Less than 50% (152/329) were included in this study. There were some differences in the full cohort versus those included in this study, but the authors did not assess whether those included in the study differed significantly from those excluded. |
| Detection bias-Exposure characterization | Definitely low risk of bias | Cord blood was measured consistently and analyzed by CDC lab. Plasma samples were conducted consistently with established methods. |
| Detection bias- Outcome characterization | Probably low risk of bias | The outcome was measured using validated instruments, but not a gold standard. |
| Selective reporting bias | Definitely low risk of bias | All measured outcomes reported |
| Other sources of bias | Probably low risk of bias | Non-linear associations were assessed, sample size may have been too small to look at effect modification. No sensitivity analyses were done. |
| **IRIS** | | |
| **Domain** | **Rating** | **Justification** |
| Exposure measurement | Good | Exposure was measured from cord blood shortly after birth. PBDE measurement was done in a CDC lab, QA/QC was performed and seemed sufficient. |
| Outcome ascertainment | Good | The Bayley Scales of Infant Development-II was used when children were 12, 24, and 36 months of age, and the WPPSI-R was used when the children were 48 and 72 months. |
| Participant selection | Deficient | Women were recruited from NYC hospitals if they were pregnant during the 9/11 attacks. They were eligible if they were between 18 and 39 years old, smoked less than one cigarette per day during pregnancy, and reported no diabetes, hypertension, HIV/AIDS, or use of illegal drugs in the preceding year. Exposure may have influenced whether women were still pregnant after the attacks and may have influenced their desire to participate in the study. |
| Confounding | Deficient | Confounders were chosen a priori and included age of child, sex of child, ethnicity, environmental tobacco smoke exposure, IQ of mother. Other covariates were included if they changed the coefficient more than 10%: gestational age at birth, maternal age, maternal education, material hardship during pregnancy, bread feeding. The language and location of the interview and assessment were included in sensitivity analyses. Consumption of fish when she was pregnant and cord blood mercury and lead concentrations were also considered as potential confounders. |
| Analysis | Good | Non-linear associations were examined and model selection was done according to form that fit the data best. Influential points were examined. |
| Selective reporting | Good | No evidence of selective reporting, all analyses described in methods were presented. |
| Sensitivity | Adequate | Range of exposure levels seems sufficient to detect an effect. The population was evaluated at a time period that would be relevant to detection of an effect. The study may not have been adequately powered to detect an effect. |
| Overall study confidence | Low | One or more deficient ratings |
| **TSCA** | | |
| **Domain** | **Rating** | **Justification** |
| Participant selection | 2 - Medium | All key elements of the study design are reported. Women were recruited if they were pregnant during the 9/11 attacks, but given that recruitment happened a few months later, there could have been selection for those with high exposure levels. |
| Attrition | 3 - Low | 152 of 329 had outcome assessed. |
| Comparison group | 1 - High | Key elements of the study design were reported and indicate that subjects were recruited from the same eligible population with the same method of ascertainment during the same time period |
| Measurement of exposure | 1 - High | Exposure was consistently measured using well-established methods that directly measure the exposure. |
| Exposure levels | 2 - Medium | The range of exposure values is sufficient to have an effect. |
| Temporality | 1 - High | Cord blood PBDEs come before child neurodevelopment at ages 12, 24, 36, 48, and 72 months, and is a relevant exposure window. |
| Outcome measurement or characterization | 2 - Medium | Well-established method was used for outcome assessment, but not a gold standard. |
| Reporting bias | 1 - High | Measured outcomes are reported in the methods, and effect estimates are reported with a confidence interval. |
| Covariate adjustment | 3 - Low | Did not include HOME inventory, marital status, alcohol use, depression |
| Covariate characterization | 1 - High | Interviews were done to get maternal self-report variables and medical records were abstracted for other variables. |
| Co-exposure confounding | 3 - Low | Study does not include discussion of potential co-exposures. |
| Study design and methods | 2 - Medium | Could not select high for this metric. The study design was appropriate and statistical methods were appropriate. |
| Statistical power | 4 - Unacceptable | Given the observed association size, the sample size was not sufficient to detect an effect for some of the measures, although not all. |
| Reproducibility of analyses | 2 - Medium | Could not select high for this metric. The description of analysis is sufficient to understand what was done and is conceptually reproducible. |
| Statistical models | 3 - Low | Decided to mark as low if model assumptions were not described. |
| Use of biomarker of exposure | 1 - High | Cord blood PBDEs have quantitative relationship with exposure. |
| Effect biomarker | NA |  |
| Method sensitivity | 3 - Low | Detection frequency was greater than 65% for only 2 of the congeners. |
| Biomarker stability | 3 - Low | No information is given about sample stability. |
| Sample contamination | 3 - Low | Decided to rate as low if sample containers were not described at all. |
| Method requirements | 1 - High | Gas chromatography/isotope dilution high-resolution mass spectrometry was done. |
| Matrix adjustment | 2 - Medium | Only lipid-adjusted results are presented. |
| Overall score | 4- Unacceptable | Rated unacceptable because at least one domain was unacceptable |

Table S12. Risk of bias ratings using the Hoffman et al. (2012) study

| **OHAT** | | |
| --- | --- | --- |
| **Domain** | **Rating** | **Justification** |
| Selection bias | Probably low risk of bias | Women were recruited from UNC prenatal care clinic, and then were followed through the first year postpartum. The next phase of the study followed children through 3 years of age. Exclusion restrictions were independent of exposure status. |
| Confounding bias | Probably high risk of bias | Did not include alcohol use, depression, income, or exposure to other neurotoxins |
| Attrition/Exclusion bias | Probably high risk of bias | Babies were only included if they were breast-fed for at least 3 months, which resulted in a sample that was more likely to be white, have higher educational attainment, and be older than the pregnancy cohort. Only 304 of original 2009 sample participated in the breast milk sample collection, and only 222 returned the behavioral assessment for their child. |
| Detection bias-Exposure characterization | Definitely low risk of bias | Exposure was measured consistently using well-established methods that directly measure exposure |
| Detection bias- Outcome characterization | Probably low risk of bias | Outcome was assessed using validated methods, but they aren't the gold standard. |
| Selective reporting bias | Definitely low risk of bias | Not all subscale results were reported in the main text, but they were reported in the supplemental material |
| Other sources of bias | Definitely low risk of bias | Non-linear associations were assessed, and the form of covariates was evaluated |
| **IRIS** | | |
| **Domain** | **Rating** | **Justification** |
| Exposure measurement | Good | Breast milk was collected at 3 months postpartum and analyzed in a CDC lab for PBDEs. |
| Outcome ascertainment | Adequate | Validated in New Haven population with limited generalizability |
| Participant selection | Deficient | Only 222 mother-child pairs had breast milk samples and therefore were included from an initial cohort size of 2,009. How many mothers were initially invited to join the cohort but declined is not reported. |
| Confounding | Deficient | Confounders included child sex and age, household income, maternal age, race, and education, parity, prenatal tobacco use, omega-3 fatty acid levels, during of breast-feeding. Covariates were chosen a priori. HOME score was measured but not included because the variability was minimal and differences were explained by income and educational attainment. |
| Analysis | Good | Non-linear associations were assessed using loess. Descriptive information about exposure and outcome presented. Describes % below LOD. Effect estimates and confidence intervals presented. |
| Selective reporting | Good | No evidence of selective reporting, all analyses described in methods were presented. |
| Sensitivity | Adequate | Range of exposure levels seems sufficient to detect an effect. The population was evaluated at a time period that would be relevant to detection of an effect. The study may not have been adequately powered to detect an effect. |
| Overall study confidence | Low | One or more deficient ratings |
| **TSCA** | | |
| **Domain** | **Rating** | **Justification** |
| Participant selection | 2 - Medium | All key elements of the study design are reported. All children were singleton births free from major birth defects. This study limited to babies who were breast-fed at least 3 months, in order to obtain a milk sample. Women were somewhat more likely to be white, have higher educational attainment, and be older compared to mothers in the full cohort. |
| Attrition | 3 - Low | 222 of 304 women returned an ITSEA evaluation for their child. Women who had outcome data were more likely to be white and have higher SES compared to those who did not. |
| Comparison group | 1 - High | Key elements of the study design were reported and indicate that subjects were recruited from the same eligible population with the same method of ascertainment during the same time period |
| Measurement of exposure | 1 - High | Exposure was consistently measured using well-established methods that directly measure the exposure. |
| Exposure levels | 2 - Medium | Could not select high for this metric. There is sufficient variation in the exposure and was observed at a relatively high level. |
| Temporality | 1 - High | Breast milk PBDE exposure precedes child neurodevelopment at ages 24-36 months. The interval is a relevant time window for exposure. |
| Outcome measurement or characterization | 2 - Medium | All key elements of the study design are reported. All children were singleton births free from major birth defects. This study limited to babies who were breast-fed at least 3 months, in order to obtain a milk sample. Women were somewhat more likely to be white, have higher educational attainment, and be older compared to mothers in the full cohort. |
| Reporting bias | 1 - High | Continuous exposure so don't need to present number of exposed / unexposed |
| Covariate adjustment | 3 - Low | Did not include marital status, alcohol use, depression, or exposure to other neurotoxins. |
| Covariate characterization | 2 - Medium | Self-administered questionnaires, telephone interview, and home visits were sue to collect information on confounders. All covariates were collected with a gold-standard method except tobacco use, which was identified using self-report and not a biomarker. |
| Co-exposure confounding | 3 - Low | Could not select high or unacceptable for this metric. No mention of co-exposures, and it is possible that phthalates could have co-occurred. |
| Study design and methods | 2 - Medium | Could not select high or low for this metric. The cohort study design was appropriate for the research design, and the statistical methods were appropriate. |
| Statistical power | 4 - Unacceptable | Could not select high or low for this metric. Sample size was large enough to detect effects for some exposure-outcome combinations, but not all. |
| Reproducibility of analyses | 2 - Medium | Could not select high or unacceptable for this metric. The description of analysis is sufficient, and is conceptually reproducible. |
| Statistical models | 3 - Low | Decided to mark as low if model assumptions were not described. |
| Use of biomarker of exposure | 1 - High | Biomarker of PBDE exposure in breast milk has quantitative relationship with exposure. |
| Effect biomarker | NA |  |
| Method sensitivity | 3 - Low | 4 congeners were detected in fewer than 70% of samples. |
| Biomarker stability | 3 - Low | Samples had a known storage history, but sample stability is not described. |
| Sample contamination | 3 - Low | There is no mention of contamination but the analysis included blanks and quality assurance. |
| Method requirements | 1 - High | Gas chromatography/isotope dilution high-resolution mass spectrometry was done. |
| Matrix adjustment | 1 - High | Wet weight and lipid-adjusted PBDE measurements were done, and patterns of association were said to be similar across methods. Only the lipid adjusted levels are reported. |
| Overall score | 4- Unacceptable | Rated unacceptable because at least one domain was unacceptable |

Table S13. Risk of bias ratings using the Lin et al. (2010) study

| **OHAT** | | |
| --- | --- | --- |
| **Domain** | **Rating** | **Justification** |
| Selection bias | Probably low risk of bias | Women were recruited from hospitals in Taiwan without knowledge of exposure levels. Mothers were enrolled based on health status and voluntary donation of cord blood and breast milk. It is possible that by requiring mothers to be healthy, they excluded mothers who had higher PBDE levels. |
| Confounding bias | Probably high risk of bias | Confounders did not include HOME inventory, maternal education, marital status, alcohol use, depression, poverty, smoking exposure, or exposure to other neurotoxic agents. |
| Attrition/Exclusion bias | Probably high risk of bias | 98 mothers were enrolled, but 35 participants were included in the analysis due to continuous breastfeeding for the first six months. |
| Detection bias-Exposure characterization | Definitely low risk of bias | Exposure was measured consistently using established methods that directly measure exposure |
| Detection bias- Outcome characterization | Probably high risk of bias | Bayley-III was used to measure infant development from 8-13 months, which is early for this test. |
| Selective reporting bias | Definitely high risk of bias | Only statistically significant results were reported. |
| Other sources of bias | Probably high risk of bias | Unclear why exposure and outcome were both analyzed as binary outcomes, and it is unclear what the cut point was for the outcome. No description of assessment of non-linearity. |
| **IRIS** | | |
| **Domain** | **Rating** | **Justification** |
| Exposure measurement | Good | PBDE measured in breast milk. No information about QA/QC. |
| Outcome ascertainment | Deficient | BSID-III was used to assess infant neurodevelopment for babies at between 8 and 13 months of age. |
| Participant selection | Deficient | Only 35 participants were included due to continuous breast-feeding from a cohort of 98 mothers. Initial recruitment process of 98 mothers not reported. |
| Confounding | Deficient | Confounders included maternal age, prepregnancy BMI, infant sex, infant age, and gestational age. |
| Analysis | Deficient | No discussion of normality distributions or assumptions. Descriptive statistics were presented. The results were presented with p-values rather than confidence intervals. Missing data not reported or addressed. |
| Selective reporting | Deficient | No evidence of selective reporting, all analyses described in methods were presented. |
| Sensitivity | Deficient | The level and variability of PBDE exposure may not have been sufficient to detect an effect. The study may not have been adequately powered to detect an effect. |
| Overall study confidence | Low | One or more deficient ratings |
| **TSCA** | | |
| **Domain** | **Rating** | **Justification** |
| Participant selection | 3 - Low | No information on inclusion criteria, or where women were recruited from beyond the fact they were clinics in Taiwan. There was also no information about how those who had continuous breast-feeding differed from the others who were not included. |
| Attrition | 3 - Low | A large portion of the 98 were missing exposure data. Only 35 had breast milk PBDEs measured. |
| Comparison group | 1 - High | Key elements of the study design were reported and indicate that subjects were recruited from the same eligible population with the same method of ascertainment during the same time period |
| Measurement of exposure | 1 - High | Exposure was consistently measured using well-established methods that directly measure the exposure. |
| Exposure levels | 4 - Unacceptable | No description is provided on the levels or range of each PBDE congener, only of the sum, and only the mean and median are presented. |
| Temporality | 1 - High | Breast milk PBDE exposure precedes child neurodevelopment at age 8-13 months. The interval is a relevant time window for exposure. |
| Outcome measurement or characterization | 3 - Low | The Bayley-III has documented limitations for children below 2 years of age. |
| Reporting bias | 3 - Low | All of the study's measured outcomes have not been reported, only those that are statistically significant are reported. |
| Covariate adjustment | 3 - Low | Did not include HOME inventory, maternal education, marital status, alcohol use, depression, poverty, exposure to tobacco smoke, exposure to other neurotoxins. |
| Covariate characterization | 3 - Low | No mention of how covariates were collected or measured. |
| Co-exposure confounding | 3 - Low | Could not select high or unacceptable for this metric. No mention of co-exposures, and it is possible that phthalates could have co-occurred. |
| Study design and methods | 4 - Unacceptable | It is unclear why logistic regression was used for a continuous outcome and exposure. They categorized PBDE levels at the 75th percentile, but it is not clear why. Also the Bayley-III scores are the outcome anad as far as I can tell they are continuous. |
| Statistical power | 4 - Unacceptable | The sample size is quite low. Some statistically significant effects are observed; however, given the other limitations of the study it is difficult to determine whether it was adequately powered. |
| Reproducibility of analyses | 3 - Low | The description of the analysis is not quite sufficient. It is unclear which results correspond to which statistical analyses. |
| Statistical models | 3 - Low | The statistical model building process is not fully appropriate and no assumptions were discussed, so can't determine if they were met. |
| Use of biomarker of exposure | 1 - High | Biomarker of PBDE exposure in breast milk has quantitative relationship with exposure. |
| Effect biomarker | NA |  |
| Method sensitivity | 3 - Low | Limits of detection are not reported. |
| Biomarker stability | 3 - Low | Samples had unknown storage history and no stability information. |
| Sample contamination | 3 - Low | No information is given about sample contamination. |
| Method requirements | 1 - High | Gas chromatography/ high-resolution mass spectrometry was done. |
| Matrix adjustment | 2 - Medium | Breast milk was lipid-adjusted, but the study only provides results with lipid-adjusted values. |
| Overall score | 4- Unacceptable | Rated unacceptable because at least one domain was unacceptable |

Table S14. Risk of bias ratings using the Roze et al. (2009) study

| **OHAT** | | |
| --- | --- | --- |
| **Domain** | **Rating** | **Justification** |
| Selection bias | Probably low risk of bias | Cohort was of 90 white, healthy pregnant women who were randomly selected from mothers of healthy, full-term, singleton infants who lived in the northern provinces of the Netherlands. It is possible that exposure may have affected whether women had healthy, full-term infants, and therefore whether they could be included in the study. |
| Confounding bias | Probably high risk of bias | Did not control for maternal age, maternal education, marital status, alcohol use, depression, exposure to tobacco smoke, or other neurotoxic agents. |
| Attrition/Exclusion bias | Probably low risk of bias | 69 of the original 90 cohort had measurements, and only 62 of those participated in the follow up program. However, the OHC concentrations of the 7 children who did not have follow up visits were not different from those who did participate. The 69 were had measurements were randomly selected from the 90 due to financial constraints. |
| Detection bias-Exposure characterization | Definitely low risk of bias | Exposure was measured consistently using established methods that directly measure exposure |
| Detection bias- Outcome characterization | Probably low risk of bias | Outcome was assessed using validated methods, but they aren't the gold standard. |
| Selective reporting bias | Probably high risk of bias | Only significant associations shown |
| Other sources of bias | Probably low risk of bias | Non-linear associations were assessed, but correlations were computed rather than regression coefficients, not sure why. |
| **IRIS** | | |
| **Domain** | **Rating** | **Justification** |
| Exposure measurement | Adequate | Exposure measured at 35 weeks gestation; may have missed earlier preterm deliveries |
| Outcome ascertainment | Adequate | Children were assessed at ages between 5-6 years. Their cognitive outcomes were assessed using the WPPSI-R, the NEPSY-II, and AVLT. Ages 5-6 are similar with validation sample for WPPSI-R and NEPSY-II, but not AVLT. Test of Everyday Attention for Children was also administered. The child's behavioral outcomes were assessed using the CBCL and the Teacher's Report Form, and ADHD questionnaire. |
| Participant selection | Deficient | Eligible women were white, healthy pregnant women who had given birth to a healthy, full-term, singleton infant. No information given about initial recruitment process or how subset different from the whole on demographics (although randomly selected, differences due to chance are still possible). |
| Confounding | Deficient | Confounders considered included socioeconomic status, HOME inventory, and child sex. |
| Analysis | Deficient | Attention paid to distribution of variables, Kolmogorov-Smirnov test was used to determine if variables were distributed normally, and correlations were tested using either Pearson or Spearman's rank correlation depending on the result. Descriptive results presented for exposure and outcome. However, only correlations presented with p-values, no standard errors or confidence intervals |
| Selective reporting | Good | No evidence of selective reporting, all analyses described in methods were presented. |
| Sensitivity | Adequate | The level and variability of BDE exposures may not have been sufficient to detect an effect. The study may not have been adequately powered to detect an effect. |
| Overall study confidence | Low | One or more deficient ratings |
| **TSCA** | | |
| **Domain** | **Rating** | **Justification** |
| Participant selection | 1 - High | All key elements of the study design are reported, participation rate is described, and participants were randomly selected. |
| Attrition | 1 - High | Minimal loss to follow up. |
| Comparison group | 1 - High | Key elements of the study design were reported and indicate that subjects were recruited from the same eligible population with the same method of ascertainment during the same time period |
| Measurement of exposure | 1 - High | Exposure was consistently measured using well-established methods that directly measure the exposure. |
| Exposure levels | 3 - Low | The range of exposure in the population is limited. |
| Temporality | 1 - High | Maternal serum PBDE levels preceded child neurodevelopment outcomes, and prenatal exposure is considered a relevant exposure window |
| Outcome measurement or characterization | 2 - Medium | Outcome was measured using well-established methods, but they aren't the gold standard. |
| Reporting bias | 3 - Low | Only significant results are shown, and confidence intervals or standard errors not presented. |
| Covariate adjustment | 3 - Low | Did not include maternal age, marital status, alcohol use, depression, exposure to tobacco smoke |
| Covariate characterization | 3 - Low | No mention of how covariates were collected or measured. |
| Co-exposure confounding | 3 - Low | Chlorinated OHCs PCP, and thyroid hormones were assessed but not controlled for in the study of PBDEs. |
| Study design and methods | 2 - Medium | Statistical models are not inappropriate, but not traditional. Only used correlations not regression models. This was captured in metric 15. |
| Statistical power | 4 - Unacceptable | The number of participants is low. There are some statistically significant results but not for all the PBDE congeners or for all measured outcomes. |
| Reproducibility of analyses | 3 - Low | Not clear which outcomes were normally distributed and which weren't, therefore unclear which correlation approach was used for which outcomes. |
| Statistical models | 3 - Low | It is not clear why they did partial correlations rather than regression coefficients. They did note that they used Pearson correlation for normally distributed variables and Spearman's rank correlation for non-normally distributed variables |
| Use of biomarker of exposure | 1 - High | Serum PBDE levels have quantitative relationship with exposure. |
| Effect biomarker | NA |  |
| Method sensitivity | 2 - Medium | Limits of detection are low enough to detect chemicals in a sufficient portion of samples to address the research question. |
| Biomarker stability | 3 - Low | Samples have known storage history, but unknown stability |
| Sample contamination | 2 - Medium | Samples are noted to have been stored in acetone-prewashed glass tubes |
| Method requirements | 2 - Medium | Gas chromatography/ mass spectrometry was done. |
| Matrix adjustment | 2 - Medium | PBDE were lipid-adjusted, and only the lipid-adjusted results are shown. |
| Overall score | 4- Unacceptable | Rated unacceptable because at least one domain was unacceptable |

Table S15. Risk of bias ratings using the Sagiv et al. (2015) study

| **OHAT** | | |
| --- | --- | --- |
| **Domain** | **Rating** | **Justification** |
| Selection bias | Probably low risk of bias | Women were enrolled during pregnancy at the community clinics where they were receiving prenatal care. Families were also recruited through local elementary schools, churches, libraries, food banks, and community events for a second wave of the cohort. |
| Confounding bias | Probably high risk of bias | Did not include alcohol |
| Attrition/Exclusion bias | Probably high risk of bias | 337 children out of 601 women from the first cohort were included, the second cohort included 305 children. |
| Detection bias-Exposure characterization | Probably low risk of bias | For women who did have pregnancy blood samples, the exposure was well-characterized using a CDC lab. For women who didn't, their values were predicted using an algorithm. |
| Detection bias- Outcome characterization | Probably low risk of bias | Outcome was assessed using validated methods, but they aren't the gold standard. |
| Selective reporting bias | Definitely low risk of bias | All measured outcomes reported |
| Other sources of bias | Definitely low risk of bias | Effect modification was assessed, non-linearity of associations was assessed, repeated measured were appropriately handled, and examined outliers or influential observations. |
| **IRIS** | | |
| **Domain** | **Rating** | **Justification** |
| Exposure measurement | Good | For those who had exposure measured at the correct time period, procedure was done in CDC lab. |
| Outcome ascertainment | Adequate | Attention was measured using Conners Continuous Performance Test (CPT II) -- did not have enough information about validation and thus adequate rating given, intelligence was measured using the WISC-IV -- validated for the right age group, risk for ADHD was measured using the CADS-P -- validated for the right age group, and behavior was assessed using the BASC-2 (validated) and SRP. |
| Participant selection | Deficient | Pregnant women were eligible if they were at least 18 years old, less than 20 weeks gestation, Spanish- or English- speaking, qualified for low-income health insurance, and were planning to deliver at the public hospital. No information given about how many women were invited to participate, but 601 were enrolled. Only 337 children remained in the study at age 9. A second wave of recruitment was done, for children age 8 or 9 whose mothers would've qualified for the original study. 305 children were recruited this way. Also, exposure was only measured at the correct time point for a subset of participants and extrapolated / predicted for the rest. |
| Confounding | Deficient | Did not have maternal alcohol use |
| Analysis | Good |  |
| Selective reporting | Adequate | Did not report results for individual PBDEs, just the sum |
| Sensitivity | Adequate | Sufficient exposure variability at levels that seem relevant for potential effects to be observed. However, sample size may have been too small to detect effects. |
| Overall study confidence | Low | One or more deficient ratings |
| **TSCA** | | |
| **Domain** | **Rating** | **Justification** |
| Participant selection | 2 - Medium |  |
| Attrition | 2 - Medium | There was substantial loss to follow up in the first cohort, which then was supplemented with the second cohort. Prenatal exposures were imputed for the second cohort. |
| Comparison group | 2 - Medium | Didn't show differences between the two cohorts. |
| Measurement of exposure | 2 - Medium | For the first cohort, exposure was consistently measured using well-established methods that directly measure the exposure. The second cohort had exposure values imputed using machine learning. |
| Exposure levels | 2 - Medium | The range of exposure in the population is large enough to observe an effect. |
| Temporality | 1 - High | Breast milk PBDE exposure precedes child neurodevelopment at age 5-12 years. The interval is a relevant time window for exposure. |
| Outcome measurement or characterization | 2 - Medium | Outcome was measured using well-established methods, but they aren't the gold standard. |
| Reporting bias | 1 - High | All outcomes were reported, and associations with confidence intervals were presented. |
| Covariate adjustment | 3 - Low | Did not include marital status or alcohol use |
| Covariate characterization | 2 - Medium | Tobacco based on self-report. |
| Co-exposure confounding | 2 - Medium | Exposure to pesticides and DDT were assessed as potential co-exposures |
| Study design and methods | 2 - Medium | The study design was appropriate and the statistical methods were appropriate. |
| Statistical power | 4 - Unacceptable | Sample size was not sufficient to detect an effect for the given effect size for several of the outcome and individual congeners. |
| Reproducibility of analyses | 2 - Medium | The description of the analysis is sufficient to be conceptually reproducible. |
| Statistical models | 3 - Low | Decided to mark as low if model assumptions were not described. |
| Use of biomarker of exposure | 1 - High | Biomarker of PBDE exposure in maternal serum has quantitative relationship with exposure. |
| Effect biomarker | NA |  |
| Method sensitivity | 3 - Low | Cited paper does not provide the LOD. |
| Biomarker stability | 3 - Low | Sample stability and storage history were not documented. |
| Sample contamination | 3 - Low | Sample contamination was not documented. |
| Method requirements | 1 - High | Gas chromatography/isotope dilution high-resolution mass spectrometry was done. |
| Matrix adjustment | 2 - Medium | Measurements reported on serum lipid basis, all results reported are lipid-adjusted. |
| Overall score | 4- Unacceptable | Rated unacceptable because at least one domain was unacceptable |

Table S16. Risk of bias ratings using the Shy et al. (2011) study

| **OHAT** | | |
| --- | --- | --- |
| **Domain** | **Rating** | **Justification** |
| Selection bias | Probably low risk of bias | Women were recruited from hospitals in southern Taiwan. 160 were invited to join, and 95 agreed to answer survey questions. 80 donated cord blood. |
| Confounding bias | Probably high risk of bias | It is not clear whether any confounders were adjusted for, although the authors report there were no associations with PBDEs and pre-pregnancy BMI, parity, education level, or household income. |
| Attrition/Exclusion bias | Probably high risk of bias | Only 36 infants returned to the hospital for neurodevelopmental assessment. |
| Detection bias-Exposure characterization | Definitely low risk of bias | Exposure was measured consistently using established methods that directly measure exposure |
| Detection bias- Outcome characterization | Probably high risk of bias | Outcome was assessed using the Bayley-III for infants 8-12 months old |
| Selective reporting bias | Probably low risk of bias | All measured outcomes reported |
| Other sources of bias | Probably high risk of bias | There is no discussion of why bootstraps were done with the logistic regression, or why logistic regression was used for what seems to be a continuous score. There is no discussion of non-linearity, sensitivity analyses, effect modification, or confounding. |
| **IRIS** | | |
| **Domain** | **Rating** | **Justification** |
| Exposure measurement | Good | Cord blood PBDE measurements were done by Supermicro Masss Research and Technology Center, Cheng Shiu University, Taiwan. |
| Outcome ascertainment | Deficient | Bayley-III used to assess neurodevelopment among children aged 8-12 months. |
| Participant selection | Deficient | Only 36 of the 160 invited to join ultimately were included due to refusal to participate, cord blood not of sufficient volume, r did not return for neurodevelopmental assessment. |
| Confounding | Deficient | Confounders included maternal age, prepregnancy BMI, and parity. |
| Analysis | Deficient | Spearman's correlation and logistic regression were analyzed/assessed. No attention paid to non-linearity. Only point estimates and p-values were presented. No comparison of included versus excluded participants. |
| Selective reporting | Good | No evidence of selective reporting, all analyses described in methods were presented. |
| Sensitivity | Deficient | There may not have been enough variability of PBDE levels, especially for some congeners. The sample size was likely too low to detect effects. |
| Overall study confidence | Low | One or more deficient ratings |
| **TSCA** | | |
| **Domain** | **Rating** | **Justification** |
| Participant selection | 3 - Low | No information on inclusion criteria, or where women were recruited from beyond the fact they were clinics in Taiwan. |
| Attrition | 3 - Low | 36 of 54 mothers with enough cord blood came back to the hospital for neurodevelopment evaluation. The sample was limited to those with non-missing exposure or outcome measures. |
| Comparison group | 1 - High | Key elements of the study design were reported and indicate that subjects were recruited from the same eligible population with the same method of ascertainment during the same time period |
| Measurement of exposure | 1 - High | Exposure was consistently measured using well-established methods that directly measure the exposure. |
| Exposure levels | 4 - Unacceptable | Levels were too low for some of the congeners. |
| Temporality | 1 - High | Cord blood PBDEs come before child neurodevelopment at ages 8-12 months, and is a relevant exposure window. |
| Outcome measurement or characterization | 3 - Low | The Bayley-III has documented limitations for children below 2 years of age. |
| Reporting bias | 2 - Medium | All outcomes were reported, but associations were not presented with confidence intervals. |
| Covariate adjustment | 3 - Low | Did not include HOME inventory, marital status, alcohol use, depression, tobacco smoke, child sex, or other neurotoxins |
| Covariate characterization | 2 - Medium | BMI was self-report. |
| Co-exposure confounding | 3 - Low | No information is given about co-exposures. |
| Study design and methods | 2 - Medium | The study design was appropriate and the statistical methods were on the whole, appropriate. |
| Statistical power | 4 - Unacceptable | Sample size was not sufficient to detect an effect for the given effect size for several of the outcome and individual congeners. |
| Reproducibility of analyses | 3 - Low | The bootstrapping procedure was not adequately explained. |
| Statistical models | 3 - Low | The bootstrapping procedure was done apparently to reduce "statistical and sampling bias" but that's not what it does, this is inaccurate. |
| Use of biomarker of exposure | 1 - High | Biomarker of PBDE exposure in cord blood has quantitative relationship with exposure. |
| Effect biomarker | NA |  |
| Method sensitivity | 2 - Medium | Limits of detection are low enough for the majority of PBDE congeners to detect chemicals in a sufficient portion of samples to address the research question. |
| Biomarker stability | 3 - Low | Sample had known storage history, but no information is given about sample stability. |
| Sample contamination | 2 - Medium | Study notes that cord blood samples were collected in a glass bottle. |
| Method requirements | 1 - High | Gas chromatography/ high-resolution mass spectrometry was done. |
| Matrix adjustment | 2 - Medium | Only lipid-adjusted results are presented. |
| Overall score | 4- Unacceptable | Rated unacceptable because at least one domain was unacceptable |

Table S17. Risk of bias ratings using the Zhang et al. (2017) study

| **OHAT** | | |
| --- | --- | --- |
| **Domain** | **Rating** | **Justification** |
| Selection bias | Probably low risk of bias | Study enrolled 468 pregnant women during pregnancy, 389 of whom remained in the study and delivered a live born singleton. Enrollment was not likely to be differential by exposure. |
| Confounding bias | Probably high risk of bias | Did not include alcohol use, or other potentially neurotoxic agent exposure |
| Attrition/Exclusion bias | Probably high risk of bias | 239 children of the original 468 pregnant women completed the assessment at ages 5 or 8. |
| Detection bias-Exposure characterization | Definitely low risk of bias | Exposure was measured consistently using established methods that directly measure exposure |
| Detection bias- Outcome characterization | Probably low risk of bias | Outcome was assessed using validated methods, but they aren't the gold standard. |
| Selective reporting bias | Definitely low risk of bias | All measured outcomes reported |
| Other sources of bias | Definitely low risk of bias | Appropriate statistical models were used, non-linear associations were assessed, sensitivity analyses were done. |
| **IRIS** | | |
| **Domain** | **Rating** | **Justification** |
| Exposure measurement | Good | PBDEs measured by NCEH at CDC. |
| Outcome ascertainment | Good | WISC-IV and BASC-2 administered at age 8. |
| Participant selection | Adequate | 468 pregnant women were initially enrolled, 389 remained in the study, and 239 had complete chemical measures in serum and completed assessment at 5 or 8 years old, and did not have congenital anomalies. No information given about how many were invited versus ended up participating. |
| Confounding | Deficient | Covariates were selected based on the significance of their relationship with the exposure and outcome, and included age, race, education, household income, parity, marital status, smoking status, maternal fish consumption, maternal depression, maternal IQ, sex of the child, HOME score. Did not include alcohol. |
| Analysis | Adequate | Log-transformed exposures when skewness was an issue. Quantitative results were presented, as were descriptive results for exposure and outcome. Dose-response evaluated using a trend test. No discussion of missing covariates. |
| Selective reporting | Good | No evidence of selective reporting, all analyses described in methods were presented. |
| Sensitivity | Deficient | Exposure levels may not have been high enough to detect an effect for some of the BPDEs. Sample size may not have been large enough. |
| Overall study confidence | Low | One or more deficient ratings |
| **TSCA** | | |
| **Domain** | **Rating** | **Justification** |
| Participant selection | 1 - High | Key elements of the study design were reported. Possible lead paint exposure was an inclusion criterion, which may make the cohort less generalizable but allows for assessment of other potential neurotoxins. |
| Attrition | 3 - Low | 15 mothers did not have exposure measures, and 131 were lost to follow up, leading to final sample of 239 mother-child pairs. |
| Comparison group | 1 - High | Key elements of the study design were reported and indicate that subjects were recruited from the same eligible population with the same method of ascertainment during the same time period |
| Measurement of exposure | 1 - High | Exposure was consistently measured using well-established methods that directly measure the exposure. |
| Exposure levels | 3 - Low | Exposure levels were lower than NHANES and they did not present the % above the LOD. |
| Temporality | 1 - High | Maternal serum PBDE levels preceded child neurodevelopment outcomes, and prenatal exposure is considered a relevant exposure window |
| Outcome measurement or characterization | 2 - Medium | Outcome was measured using well-established methods, but they aren't the gold standard. |
| Reporting bias | 3 - Low | The externalizing behavior has subscales that are not reported. |
| Covariate adjustment | 3 - Low | Did not include alcohol use |
| Covariate characterization | 2 - Medium | Tobacco and fish were based on self-report. |
| Co-exposure confounding | 2 - Medium | Measured maternal blood lead levels and examined as potential co-exposure. |
| Study design and methods | 2 - Medium | Study design was appropriate, as were statistical methods. |
| Statistical power | 4 - Unacceptable | Some of the congeners had enough sample size to detect a significant effect, but not all. |
| Reproducibility of analyses | 2 - Medium | The description of the analysis is sufficient to be conceptually reproducible. |
| Statistical models | 3 - Low | Model assumptions were not described, so cannot assess whether they were met. |
| Use of biomarker of exposure | 1 - High | Serum PBDE levels have quantitative relationship with exposure. |
| Effect biomarker | NA |  |
| Method sensitivity | 3 - Low | Only 4 of the 10 PBDEs were detected in at least 90% of the samples. |
| Biomarker stability | 3 - Low | Samples have known storage history, but unknown stability |
| Sample contamination | 3 - Low | Sample contamination was not documented. |
| Method requirements | 1 - High | Gas chromatography/isotope dilution high-resolution mass spectrometry was done. |
| Matrix adjustment | 2 - Medium | Only lipid-adjusted results are presented. |
| Overall score | 4- Unacceptable | Rated unacceptable because at least one domain was unacceptable |
